# CDH17-targeting CAR-NK cells synergize with CD47 blockade for potent suppression of gastrointestinal cancers

**DOI:** 10.1016/j.apsb.2025.03.039

**Published:** 2025-03-19

**Authors:** Liuhai Zheng, Youbing Ding, Xiaolong Xu, Huifang Wang, Guangwei Shi, Yang Li, Yuanqiao He, Yue Gong, Xiaodong Zhang, Jinxi Wei, Zhiyu Dong, Jiexuan Li, Shanchao Zhao, Rui Hou, Wei Zhang, Jigang Wang, Zhijie Li

**Affiliations:** a Department of Critical Care Medicine, Guangdong Provincial Clinical Research Center for Geriatrics, Shenzhen Clinical Research Centre for Geriatrics, Department of Nuclear Medicine, Shenzhen People's Hospital (the First Affiliated Hospital, Southern University of Science and Technology, the Second Clinical Medical College, Jinan University), Shenzhen 518020, China; b Integrated Chinese and Western Medicine Postdoctoral Research Station, Jinan University, Guangzhou 510632, China; c Department of Medical Imaging, the Third Affiliated Hospital of Southern Medical University (Academy of Orthopedics Guangdong Province), Guangzhou 510630, China; d Department of Neurosurgery & Medical Research Center, Shunde Hospital, Southern Medical University (the First People's Hospital of Shunde Foshan), Guangzhou 510515, China; e Center of Laboratory Animal Science, Nanchang University, Nanchang 330031, China; f Key Laboratory of New Drug Evaluation and Transformation of Jiangxi Province, Nanchang 330031, China; g Department of Urology, the Fifth Affiliated Hospital, Southern Medical University, Guangzhou 510900, China; h Department of Urology, the Third Affiliated Hospital of Southern Medical University, Guangzhou 510630, China; i Department of Urology, Nanfang Hospital, Southern Medical University, Guangzhou 510515, China; j Center for Drug Research and Development, Guangdong Provincial Key Laboratory for Research and Evaluation of Pharmaceutical Preparations, Guangdong Pharmaceutical University, Guangzhou 510006, China; k State Key Laboratory for Quality Ensurance and Sustainable Use of Dao-di Herbs, Artemisinin Research Center, Institute of Chinese Materia Medica, China Academy of Chinese Medical Sciences, Beijing 100700, China; l Department of Oncology, the Affiliated Hospital of Southwest Medical University, Luzhou 646000, China; m Department of Traditional Chinese Medicine and School of Pharmaceutical Sciences, Southern Medical University, Guangzhou 510515, China; n State Key Laboratory of Antiviral Drugs, School of Pharmacy, Henan University, Kaifeng 475004, China

**Keywords:** Gastrointestinal cancers, CDH17, Nanobody, CAR-NK, CD47–SIRP*α* checkpoint, CV1, Macrophage activation, M1-phenotype macrophages

## Abstract

Gastrointestinal (GI) cancers are a leading cause of cancer morbidity and mortality worldwide. Despite advances in treatment, cancer relapse remains a significant challenge, necessitating novel therapeutic strategies. In this study, we engineered nanobody-based chimeric antigen receptor (CAR) natural killer (NK) cells targeting cadherin 17 (CDH17) for the treatment of GI tumors. In addition, to enhance the efficacy of CAR-NK cells, we also incorporated CV1, a CD47–SIRP*α* axis inhibitor, to evaluate the anti-tumor effect of this combination. We found that CDH17-CAR-NK cells effectively eliminated GI cancers cells in a CDH17-dependent manner. CDH17-CAR-NK cells also exhibit potent *in vivo* anti-tumor effects in cancer cell-derived xenograft and patient-derived xenograft mouse models. Additionally, the anti-tumor activity of CDH17-CAR-NK cells is synergistically enhanced by CD47–signal regulatory protein *α* (SIRP*α*) axis inhibitor CV1, likely through augmented macrophages activation and an increase in M1-phenotype macrophages in the tumor microenvironment. Collectively, our findings suggest that CDH17-targeting CAR-NK cells are a promising strategy for GI cancers. The combination of CDH17-CAR-NK cells with CV1 emerges as a potential combinatorial approach to overcome the limitations of CAR-NK therapy. Further investigations are warranted to speed up the clinical translation of these findings.

## Graphical abstract

CDH17-targeted CAR-NK cells combined with CV1 demonstrate enhanced anti-tumor activity in GI cancers by promoting macrophage activation and increasing M1 macrophage numbers, offering a promising therapeutic strategy.

**Figure:**
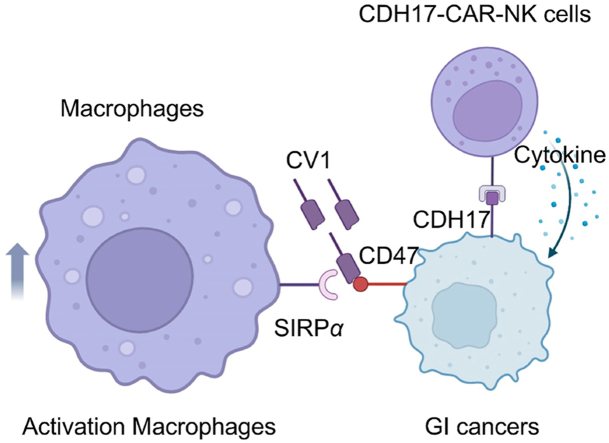

## 1 Introduction

Gastrointestinal (GI) cancers, including colorectal cancer (CRC), gastric cancer (GC), liver cancer, pancreatic cancer (PC), and esophageal cancer, are a leading cause of cancer morbidity and mortality worldwide^1^. Conventional therapies for GI cancers, such as chemotherapy, surgery, and radiation, alone or in combination, have not yielded sufficient overall survival rates^1^. Recently, various immunotherapies for GI cancers have been developed, including but not limited to checkpoint inhibitors, cytokines, vaccines, and adoptive T cell transfer therapies^2^. For example, PD1 blockades have shown significant efficacy in esophageal cancers characterized by a high burden of somatic mutations^3^. Checkpoint inhibitors have also demonstrated robust anti-tumor effects in clinical trials for hepatocellular carcinoma^4^. Despite progress in immunotherapy, the options for effectively treating patients with advanced disease remain limited, and surgical resection remains the primary intervention for patients^5^. PD1 blockades were effective in only a small subset of patients with PDAC^6^. The combination of the two most promising vaccines, GVAX and CRS-207, for the treatment of PDAC also had a low clinical response^7^. Hence, there is an urgent need for innovative therapeutic strategies to improve the survival of patients with GI cancers.

Chimeric antigen receptor-engineered natural killer (CAR-NK) cells provide a new opportunity for the treatment of GI cancers. NK cells, as natural immune cells, can eliminate malignantly transformed cells without major histocompatibility complex (MHC) restriction^8^. Unlike CAR-T cell therapy, CAR-NK cell therapy has not been associated with cytokine release syndrome (CRS) or immune effector cell-associated neurotoxicity syndrome (ICANS), irrespective of the use of autologous or allogeneic NK cells^9^. One possible reason for these advantages could be that NK cells do not proliferate and secrete cytokines as efficiently as T cells do *in vivo*^10^. Furthermore, the relatively short lifespan of CAR-NK cells leads to their rapid depletion post-tumor cell eradication, obviating the need for suicide vectors^11^. These cells can be efficiently mass-produced *in vitro*, making allogeneic transplantation feasible and reducing costs. Several studies have demonstrated the effectiveness of genetically modified fresh NK cells or the NK92MI cell lines with CARs specific to tumor antigens in various cancer models^12^^,^^13^. Though these recent breakthroughs present an intriguing opportunity for targeting and eradicating GI cancers using CAR-NK cells, the development of such therapies for GI cancers has yet to materialize. This is primarily due to the limited availability of appropriate cell surface antigens specific to GI cancers and the corresponding antibodies necessary for CAR construction.

Cadherin 17 (CDH17), also known as liver intestine (LI)-cadherin, is a calcium-dependent transmembrane glycoprotein that plays a crucial role in intercellular adhesion and maintaining tissue integrity through the regulation of the intercellular cleft in a Ca^2+^-dependent manner^14^. Under physiological conditions, CDH17 expression is primarily confined to epithelial cells in the small intestine and colon, sparing vital organs^15^. However, its expression becomes upregulated in GC^16^, CRC^17^, hepatocarcinoma (HCC)^18^, and PC^15^. Elevated CDH17 expression has been linked to tumor progression, invasion, metastasis, and poor prognosis in GC patients^19^. Consequently, various therapeutic formats targeting CDH17 have shown favorable outcomes in pre-clinical settings, including CDH17 antibodies coupled with toxins such as saporin^20^ and TRAIL^17^, as well as CDH17 CAR T cells^17^. Additionally, our previous studies have also shown that CDH17 targeting nanobody-engineered extracellular vesicles or the toxin PE38 exhibits a robust antitumor effect on GI cancers^21^^,^^22^. Thus, CDH17 is a promising target for the treatment of GI cancers. However, it remains unclear whether targeting CDH17 through CAR-NK can confer benefits with GI cancers.

CD47, recognized as a transmembrane protein, is highly expressed on both normal and cancer cells. It engages with signal regulatory protein *α* (SIRP*α*) on macrophages, which inhibits their phagocytosis of normal cells and, in turn, aids tumor cells in evading the immune system^23^. Blocking CD47 with agents such as CV1 can enhance macrophage function to engulf tumor cells, making it a promising strategy for cancer therapy^23^. However, CD47-targeted monotherapy has shown limited efficacy against solid tumors, prompting the development of combination therapies with other immunotherapies to enhance antitumor effects^24^^,^^25^. CV1, a high-affinity CD47 blocker, demonstrates minimal therapeutic effects when used alone but exhibits potential when combined with treatments like therapeutic antibodies, chemotherapy, and adoptive cell transfer, such as CAR-T cells^23^^,^^26^. This combination approach has been shown to exert significant antitumor activity, suggesting that the synergistic activation of immune cells may be key to overcoming the immunosuppressive tumor environment. However, the potential of combining CAR-NK cell therapy with CD47 blockade has not yet been extensively explored.

Herein, we developed CDH17 Nb-engineered CAR-NK cells. CDH17 Nb was identified through screening a naïve nanobody phage display library against domains 1–3 of the human CDH17 protein, exhibiting high binding affinity to both human and mouse CDH17 in our laboratory (Patent number: ZL202111648151.4 and ZL202111305763.3). Our findings demonstrate that CDH17-targeting CAR-NK cells effectively eliminate multiple GI cancers in both *in vitro* cell models and *in vivo* tumor models, including cell-derived xenograft (CDX) and patient-derived xenograft (PDX). To augment the anti-tumor function of CAR-NK cells, we combine a high-affinity CD47 blocker, CV1^23^, with CDH17-targeting CAR-NK cells. This combination has previously shown a significant enhancement in the anti-tumor effect of CAR-T cells^25^. Our results demonstrate that CV1 can significantly improve the anti-tumor effects of CDH17-targeting CAR-NK cells. Additionally, we explore the possible mechanisms underlying the synergistic effect of CDH17-targeting CAR-NK cells with CV1. Specifically, CV1 was found to enhance the activation of macrophages and increase the M1-phenotype macrophages in the tumor microenvironment, likely representing one of the key mechanisms. Our study provides a compelling strategy to amplify the therapeutic potential of CDH17-targeting CAR-NK cells through the combination with CV1 for clinical applications in GI cancer therapy.

## 2 Materials and methods

### 2.1 Antibodies and reagents

CDH17 (#A5286) was purchased from Abclonal (Wuhan, China). HA tag Rabbit mAb (#3724) was purchased from Cell Signaling Technology (CST, Danvers, MA, USA). Alexa Fluor® 647-IgG H&L (#ab150075) was purchased from Abcam (Cambridge, MA, USA). Anti-Camelid VHH Cocktail-iFluor 647 (A02019) was purchased from Kingsray Biotechnology Co., Ltd. (Nanjing, China). Fetal bovine serum (#10270106) and horse serum (#26050088) were purchased from Gibco BRL (Grand Island, NY, USA). Luria–Bertani (LB) broth (#L8291) was purchased from Solarbio Life Sciences Co., Ltd. (Beijing, China). Inositol (#I7508-50G) and folic acid (#F8758-5G) were purchased from Sigma–Aldrich (St. Louis, MO, USA). LDH-Cytox Assay Kit (#426401) was purchased from BioLegend (San Diego, CA, USA). Lenti-X Concentrator (#631231) was purchased from Clontech (Tokyo, Japan). The granzyme B (#1118502) and TNF*α* ELISA kit (#1117202) were purchased from Dakewe Biotech (Shenzhen, China). IFN*γ* ELISA kit (#EK0373) was purchased from Boster Biological Technology (Wuhan, China). For immunofluorescence assays, primary antibodies were utilized at a dilution of 1:100, while secondary antibodies were applied at a dilution of 1:1000 unless stated otherwise.

### 2.2 Cell lines and cell culture

ASPC1, MKN45, IM95, NK92, and 293T/17 were procured from Procell Life (Wuhan, China). MKN45 and ASPC1 cells were cultured in RPMI1640 supplemented with 10% fetal bovine serum (FBS), 2 mmol/L l-glutamine, and 1% penicillin/streptomycin. 293T/17 and IM95 were maintained in DMEM supplemented with 10% FBS, 2 mmol/L l-glutamine, and 1% penicillin/streptomycin. NK92 cells were cultured in alpha MEM supplemented with 0.1 mmol/L 2-mercaptoethanol, 0.02 mmol/L folic acid, 0.2 mmol/L inositol, and 100 U/mL recombinant IL-2, adjusting to a final concentration of 12.5% fetal bovine serum (FBS) and 12.5% horse serum. All cell lines were maintained at 37 °C in a humidified 5% CO_2_ incubator.

### *2.3* *CDH17* gene *profile from public cancer patient databases*

An analysis of *CDH17* gene expression between cancerous and non-tumor tissues was carried out using the gene expression profiling interactive analysis 2 (GEPIA2) website (http://gepia.cancer-pku.cn/). GEPIA2 offers data visualization tools for analyzing RNA-Seq expression data sourced from the cancer genome atlas (TCGA) and the genotype-tissue expression (GTEx) projects. The data screening conditions included matching normal data from TCGA and GTEx, selecting mRNA as the data type, and employing the LIMMA method for differential analysis. The |Log_2_FC| Cutoff and *q*-value Cutoff were set at 1 and 0.01, respectively.

### 2.4 Single-cell RNA-seq analysis

We first downloaded 10X scRNA-seq data of PDAC samples from the GSE156405^27^, GC samples from the GSE163558^28^, and CRC samples from the GSE188711^29^ in Gene Expression Omnibus (GEO) (https://www.ncbi.nlm.nih.gov/). These three datasets were processed separately using a Python-based toolkit, Scanpy (version 1.8.2)^30^. The expression matrix was normalized using raw UMI counts, which were first adjusted for total counts per cell (library size) and then scaled and transformed logarithmically. The analysis focused on selecting the top 2000 genes exhibiting high variability for subsequent examination.

Dimensionality reduction and unsupervised clustering were conducted following the standard workflow implemented in Scanpy. Principal Component Analysis (PCA) was applied to the top 2000 highly variable genes to mitigate noise. Then, the Harmony algorithm was used to correct the potential batch effects, and the top 20 principal components were used for graph construction. A k-nearest neighbor (KNN) graph was created using BBKNN^31^, utilizing Euclidean distance within the harmony-corrected PCA space. The edge weights between cells were further adjusted based on the degree of shared overlap within their local neighborhoods. Leiden algorithm^32^ was applied to the resulting KNN graph to detect cellular clusters. The Uniform Manifold Approximation and Projection (UMAP) was generated for visualization by utilizing the top 20 principal components for non-linear dimension reduction. Cell clusters were annotated based on well-known cell markers reported in the original literature for each dataset^27^, ^28^, ^29^. To identify malignant cells, we assessed CNV profiles in each dataset based on the reasoning that epithelial cells with a high degree of CNVs are likely to be malignant. We used a sliding window approach executed in the infercnvpy package (https://github.com/broadinstitute/inferCNV) to obtain CNV profiles of all cells^33^. Once all the cell clusters were annotated, we could examine the gene expression patterns of *CDH17* across different cell types in each dataset.

### 2.5 Immunofluorescence staining

For the surface detection of CDH17, cells underwent twice PBS washes, followed by fixation in 4% paraformaldehyde for 5 min. Subsequently, cells were treated with 4% donkey serum solution for 1 h at room temperature. The primary antibody, appropriately diluted in cold blocking buffer, was then applied to incubate for 1 h at room temperature. After with 3 times PBS washes, cells were incubated with a secondary antibody. To detect CAR expression on the NK92 cell surface, the anti-VHH AF 647-conjugated secondary antibody (diluted 1/1000) was applied in cold blocking buffer for 30 min. Following a 20-min fixation in 4% paraformaldehyde, cells were stained with DAPI in PBS for 20 min and mounted on slides. All staining procedures were examined and captured using a Leica SP8 laser scanning confocal microscope.

### 2.6 Plasmid construction and lentivirus production

The second-generation CARs, incorporating a CD8 signal peptide, VHH nanobody, CD8 hinge, CD28 transmembrane domain, CD28 co-stimulatory domain, and CD3*ζ* domain, were inserted into the pCD513B-CopGFP lentiviral vector by GENEWIZ (Suzhou, China). This bicistronic vector was connected to the transduction marker CopGFP through the “self-cleaving” T2A peptide. Lentivirus production involved co-transfecting HEK293T/17 cells with pMD2.G, psPAX2, and the relevant plasmids (CDH17-CAR, C9-CAR). Viral supernatants were harvested at 48 and 72 h and then concentrated using the Lenti-X Concentrator. The concentration steps followed the manufacturer's instructions in detail.

### 2.7 Generation of CAR-NK cells

NK92 cells were transfected with CDH17-CAR or C9-CAR lentivirus. Following 48 h, the cell culture medium received supplementation with the selection antibiotic puromycin (2.5 μg/mL), and a 10-day selection period with puromycin ensued. To confirm the expression of CAR on the surface of NK92 cells, cells were stained with anti-VHH-AF647 antibody and analyzed using flow cytometry. CAR-expressing NK92 cells were sorted by FACS using the SH800ZBP sorter. Post-sorting, the collected positive cells were rinsed with prewarmed medium and then cultured for subsequent use.

### 2.8 Flow cytometry analysis

CAR-modified NK92 cells were incubated with anti-VHH-AF647 antibody in FACS staining buffer (PBS with 2% FBS) on ice for 30 min for staining. Following this, cells were washed with PBS and analyzed on a DxFLEX flow cytometer (Beckman Coulter, Miami, FL, USA). For the analysis of tumor-infiltrating immune cells, ASPC1-bearing mice were treated according to the schedule and euthanized on Day 16. Tumors were harvested, mechanically minced, and then digested (tissue enzymatic digestion kit, RWD Life Science Co., Ltd., Sugar Land, TX, America) in an incubator for 45 min at 37 °C. Samples were filtered, washed, lysed in ACK buffer, and counted for flow cytometry analysis. A 100 μL single-cell suspension (∼1 × 10^6^ cells) was incubated with anti-mouse CD16/CD32 antibody (Bioxcell, clone 2.4G2, BE0307), followed by incubation with the following fluorochrome-labeled antibodies at 4 °C for 30 min: CD45-PECF594 (BD Biosciences, 562420), Gr1-PE (Biolegend, 108408), F4/80-APC (Biolegend, 123116), CD69-Brilliant Violet 421 (Biolegend, 104528), CD86-APC-Cy7 (Biolegend, 105030). After washing, the antibody-stained cells were further stained with ViaDye Red to identify dead cells. Finally, the cells were detected using Cytek Aurora spectral flow cytometry. Flow data were then analyzed using FlowJo 10 (Tree star Inc., Ashland, OR, USA).

### 2.9 *In vitro* cytotoxicity assays

To assess the cytotoxic activity of CAR-NK cells, the lactate dehydrogenase (LDH) release assay was employed. Briefly, 2 × 10^5^ target cells (IM95 and ASPC1) were co-incubated with CAR-NK cells for 4 h at 3 different Effector:Target (E:T) ratio. The release of LDH in the supernatants was assessed following the manufacturer's instructions. The levels of granzyme B, IFN-*γ*, and TNF-*α* were assessed with a commercial ELISA kit. The percentage of cytotoxicity was calculated as Eq. (1):

(1) Percentage of cytotoxicity (%)=(CAR-NK treatment release–Spontaneous release)/(Positive lysate release–Spontaneous release) × 100

### 2.10 Immunohistochemistry

Immunohistochemistry was performed to analyze and assess the expression level of CDH17 in both PDX tumor tissues and clinical samples. The PDX tissue was derived from an individual diagnosed with stage III C gastric adenocarcinoma. Clinical specimens were generously provided by Dr. Yuanqiao He. Patient consent was obtained, and all procedures involving human samples were ethically approved by the medical ethics committees (The Second Affiliated Hospital of Nanchang University). The tissue sections underwent deparaffinization and were followed by antigen retrieval in Tris–EDTA buffer (pH 9.0) at 100 °C for 15 min. Following blocking (5% BSA and 0.05% Tween-20 in PBS) for 1 h at room temperature, the sections were subjected to overnight incubation with the primary mouse anti-CDH17 antibody at 4 °C. CDH17 detection was conducted using a biotin-conjugated secondary antibody and an ABC kit. Images were captured using a 3DHISTECH scanner (Sysmex, Milton Keynes, UK). The staining scoring of samples was automated using the IHC Profiler plugin in Image J.

### 2.11 Bio-orthogonal labeling of CAR-NK cells

CAR-NK cells were cultured with Ac_4_ManNAz at a final concentration of 20 μmol/L for 48 h at 37 °C. Following the incubation, the cells were thoroughly washed 3 times with PBS to eliminate any unbound Ac_4_ManNAz, ensuring that only the incorporated label remained within the cells. The cells were then resuspended in a fresh culture medium and incubated with ICG-DBCO, a fluorescent dye conjugated to a bio-orthogonal reactive group, at a final concentration of 40 μmol/L for 1 h at 37 °C. After the labeling process, the cells were washed twice with PBS to remove any unreacted ICG-DBCO and then counted for further use.

### 2.12 Animal studies

All animal experiments were carried out with the approval of the Institutional Animal Care and Use Committee of Shenzhen People's Hospital and in accordance with the guidelines (approval No. AUP-220101-LZJ-0600-01). Six-week-old male NCG mice (*Prkdc*^–^*Il2rg*^–^) were obtained from GemPharmatech (Guangdong, China) and maintained under standard conditions, including a room temperature of approximately 25 °C and a 12-h light–dark cycle. To establish IM95 and ASPC1 xenograft model, NCG mice were injected subcutaneously with 5 × 10^6^ for IM95 cells and 3 × 10^6^ for ASPC1 cells in 100 μL PBS. When the tumor volume reached around 50 mm^3^, mice were randomly divided into six groups (five mice per group for IM95 and six mice per group for ASPC1). CDH17-CAR-NK92 cells or C9-CAR-NK92 cells (1 × 10^7^ cells) were given once every 4 days through the tail vein, for a total of 4 injections. In the PDX model, the PDX tissue was swiftly dissected into fragments on ice and then implanted subcutaneously into the right forelimbs of NCG mice. When tumor volume reached around 50 mm^3^, mice were randomly divided into three groups (4–5 mice per group). CDH17-CAR-NK92 cells or C9-CAR-NK92 (1 × 10^7^ cells) were given once every 4 days through the tail vein, for a total of 4 injections. Animal weight and tumor volume were collected every 2 days. The tumor size was calculated as Eq. (2):

(2) Tumor size (mm^3^) = Length × Width^2^/2

When the tumor burden reached 2000 mm^3^ or the animal's weight was reduced by ≥ 15%, mice were euthanized with carbon dioxide narcosis. The carbon dioxide flow rate was initially set at 1.2 L/min, corresponding to approximately 30% of the chamber volume exchange rate per minute, and was gradually increased to 2.8 L/min, achieving a 70% volume exchange rate per minute. This approach aligns with recommended euthanasia guidelines to minimize distress. Alternatively, some mice were deeply anesthetized with isoflurane before cervical dislocation, depending on procedural requirements for different experiments. Post-mortem examinations, including histopathological assessments with hematoxylin and eosin (H&E) staining, were performed on animals euthanized by both carbon dioxide asphyxiation and deep anesthesia followed by cervical dislocation to evaluate potential pulmonary hemorrhage. The findings, presented in Supporting Information Fig. S1, revealed no significant evidence of pulmonary hemorrhage or other signs of compromised animal welfare. Survival data for the animals were recorded concurrently. Additionally, efforts were made to ensure humane endpoints were strictly followed in compliance with institutional and regulatory ethical guidelines for animal research.

### 2.13 Statistical analysis

All data were analyzed using GraphPad Prism 8 software. The results were presented as mean ± standard error (SEM). Group differences were assessed using two-way analysis of variance (ANOVA) followed by Tukey's multiple comparison test or unpaired *t*-test analysis, as appropriate. Statistical significance was defined as ∗*P* < 0.05, ∗∗*P* < 0.01, ∗∗∗*P* < 0.001, and ∗∗∗∗*P* < 0.0001.

## 3 Results

### 3.1 CDH17 is highly expressed in PDAC, GC, and CRC

**Figure 1:**
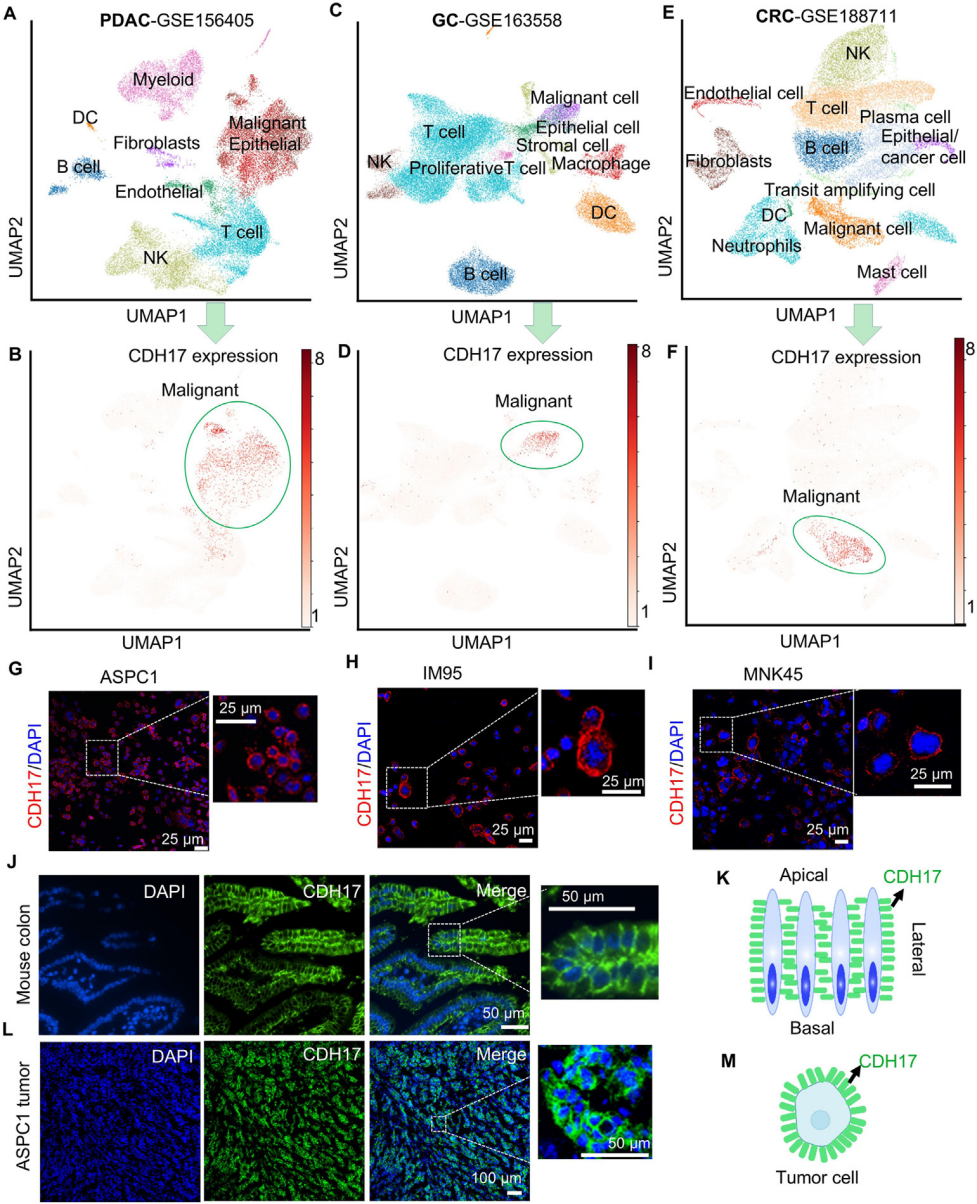
Cadherin 17 (CDH17) expression patterns in gastrointestinal (GI) cancers and colon epithelial cells. (A) The uniform manifold approximation and projection (UMAP) visualization of all cells of pancreatic ductal adenocarcinoma (PDAC) dataset. (B) The expression of *CDH17* in different cell subpopulations in the PDAC dataset. (C) The UMAP visualization of all cells in the gastric cancer (GC) dataset. (D) The expression of *CDH17* in various cell subpopulations within the GC dataset. (E) The UMAP visualization of all cells in colorectal cancer (CRC) samples. (F) The expression of *CDH17* across different cell subpopulations in the CRC dataset. (G–I) Immunofluorescence staining showing CDH17 expression on the cell surface of PAAD cell line ASPC1 (G) and STAD cell line IM95 (H) and MKN45 (I). (J) Immunofluorescence staining of CDH17 in mouse colon tissue. (K) Schematic representation depicting the expression localization of CDH17 in the mouse colon. (L) Immunofluorescence staining of CDH17 in ASPC1 tumor tissues. (M) Schematic representation depicting the expression localization of CDH17 on the ASPC1 tumor cells.

To ascertain the suitability of CDH17 for CAR-NK therapy targeting PDAC, GC, and CRC, we first performed single-cell RNA-seq (scRNA-seq) data analysis using three different datasets (GSE156405, GSE163558, and GSE188711) and annotated all cells based on the original studies (Fig. 1A–C, and E). As expected, CDH17 expression was predominantly observed in malignant cells across all datasets of these three GI cancer types (Fig. 1B–D, and F). These results suggest CDH17 is a common marker of the malignant cells in GI cancers. Additionally, we confirmed CDH17 expression on the cell surface of stomach adenocarcinoma (STAD-IM95 and MKN45) and pancreatic adenocarcinoma (PAAD-ASPC1) cell lines (Fig. 1G–I). We also performed mouse colon CDH17 immunostaining and confirmed robust expression of CDH17, predominantly localized to the lateral side of the epithelial cells in the mouse small intestine, rather than the apical or basal side^34^ (Fig. 1J and K). This lateral localization may pose challenges for CAR-NK cells in reaching or binding to CDH17 at the tight junction between intestinal epithelial cells. Additionally, we performed immunofluorescence staining for CDH17 in the subcutaneous tumors of mice bearing ASPC1 cells (a human pancreatic cancer cell line), and the results showed substantial CDH17 protein expression around the tumor cells (Fig. 1L and M) We also analyzed the expression level of CDH17 between normal tissues (N) and tumor samples (T) across multiple GI cancer types by GEPIA2 website^35^. As shown in Supporting Information Fig. S2, a significant upregulation of CDH17 is observed in tumor tissues across PDAC, GC, and CRC. Collectively, our findings support CDH17 as a promising target for CAR-NK therapy in PDAC, GC, and CRC.

### 3.2 Generation and *in vitro* assessment of CAR-NK cells

**Figure 2:**
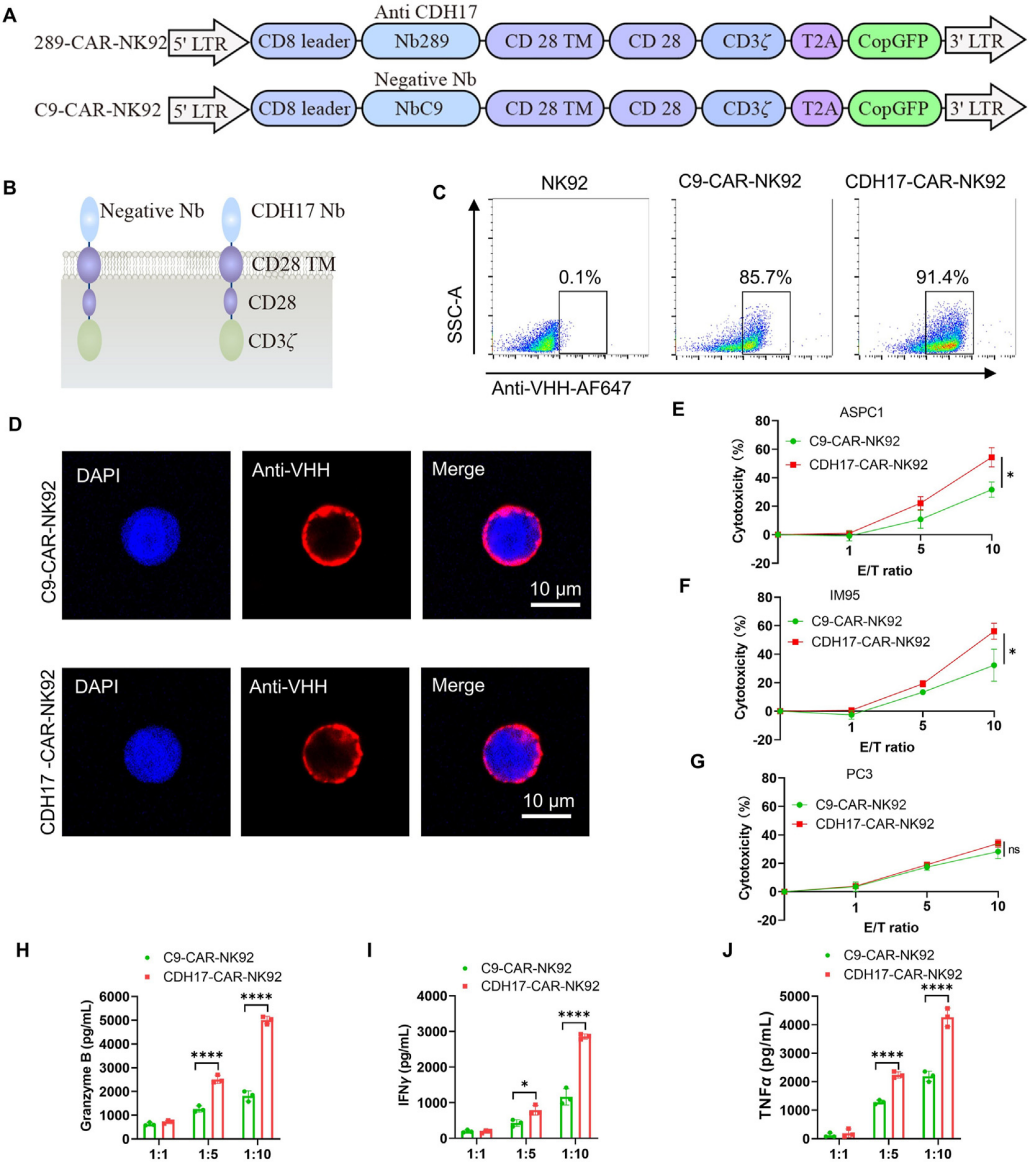
Generation and characterization of CDH17 targeted CAR-NK92 cells. (A) Schematic diagram of CAR-NK92 with an anti-CDH17 nanobody (NbCDH17) or an irrelevant nanobody (NbC9), a transmembrane region (CD28), followed by the intracellular domains of co-stimulatory CD28, and the intracellular domain of CD3*ζ*. (B) Structure schematic of the CAR-NK92 cells based on CDH17 or C9 nanobody. (C) Flow cytometry analysis of CAR expression on the surface of C9-CAR-NK92 and CDH17-CAR-NK92 cells (*n* = 3). (D) Immunofluorescence staining for CAR expression on the cell surface in C9-CAR-NK92 and CDH17-CAR-NK92 cells. (E, F) LDH released-based cytotoxicity assay of CDH17-CAR-NK92 cells or control C9-CAR-NK92 cells against ASPC1 (E) and IM95 (F) cells at three different E:T ratios (*n* = 3). IM95 and ASPC1 cells were co-incubated with CAR-NK cells for 4 h. (G) Cytotoxicity assay in CDH17 negative PC3 cells with CDH17-CAR-NK92 cells or control C9-CAR-NK92 cells. (H–J) ELISA of granzyme B (H), interferon-gamma (IFN*γ*) (I), and tumor necrosis factor-alpha (TNF*α*) (J) released after co-culture C9-CAR-NK92 cells or CDH17-CAR-NK92 cells with ASPC1 (*n* = 3). Data are presented as mean ± SEM from 3 independent experiments. *P* values were calculated based on the unpaired Student's *t*-test. ns, no significance; ∗*P* < 0.05, ∗∗∗∗*P* < 0.0001.

To target CDH17 cell surface expression cells, CDH17-CAR-NK cells were constructed using the CDH17 target nanobody with high binding affinity to both human and mouse CDH17 protein (Supporting Information Fig. S3). An irrelevant nanobody (NbC9)was used to construct negative control CAR NK cells. The CAR backbone consisted of a CD8 hinge and a transmembrane region (CD28), followed by the intracellular domains of co-stimulatory CD28 and the intracellular domain of CD3*ζ* activation domain. The CARs were cloned into the upstream of a T2A sequence and CopGFP in a lentiviral vector (Fig. 2A and B). After transduction, C9-CAR-NK92 cells and CDH17-CAR-NK92 cells were selected *in vitro* for 10 days in medium containing puromycin (2.5 μg/mL), obtaining stable C9-CAR and CDH17-CAR molecule expression. After sorting by flow cytometry with anti-VHH antibody, the percentage of C9-CAR and CDH17-CAR positive NK92 cells were 85.7% and 91.4% respectively (Fig. 2C). The high level of CARs expression was also confirmed by immunofluorescence analysis with VHH antibody (Fig. 2D). Meanwhile, the CDH17 level on the cell surface of CDH17-CAR-NK92 cells was analyzed by immunofluorescence staining, and no obvious fluorescence signal was detected, indicating no potential fratricide effect among CDH17-CAR-NK92 cells (data not shown).

We next evaluated the capacity of CDH17 targeted CAR-NK92 cells to eradicate CDH17 positive cell lines (IM95 and ASPC1) at 3 different E:T (effector-to-target) ratios. The results showed that CDH17-CAR-NK92 cells showed significant killing activity against both ASPC1 and IM95 cells at E:T cell ratios of 10:1 compared to C9-CAR-NK92 cells (Fig. 2E and F). In addition, there was no difference in the killing ability of C9-CAR-NK92 cells and CDH17-CAR-NK92 cells against the CDH17 surface-negative cancer cell line PC3, suggesting that CDH17-CAR-NK92 kill tumor cells by specifically targeting CDH17 (Fig. 2G). Consistently, CDH17-CAR-NK92 cells also released more granzyme B, IFN*γ*, and TNF*α* than C9-CAR-NK92 cells when co-cultured with ASPC1 (Fig. 2H–J). Collectively, these results demonstrate that targeting CDH17-CAR-NK cells is successfully established and exhibits prominent *in vitro* anti-tumor performance.

### 3.3 CDH17-targeting CAR-NK cells significantly repress GC tumor growth *in vivo*

**Figure 3:**
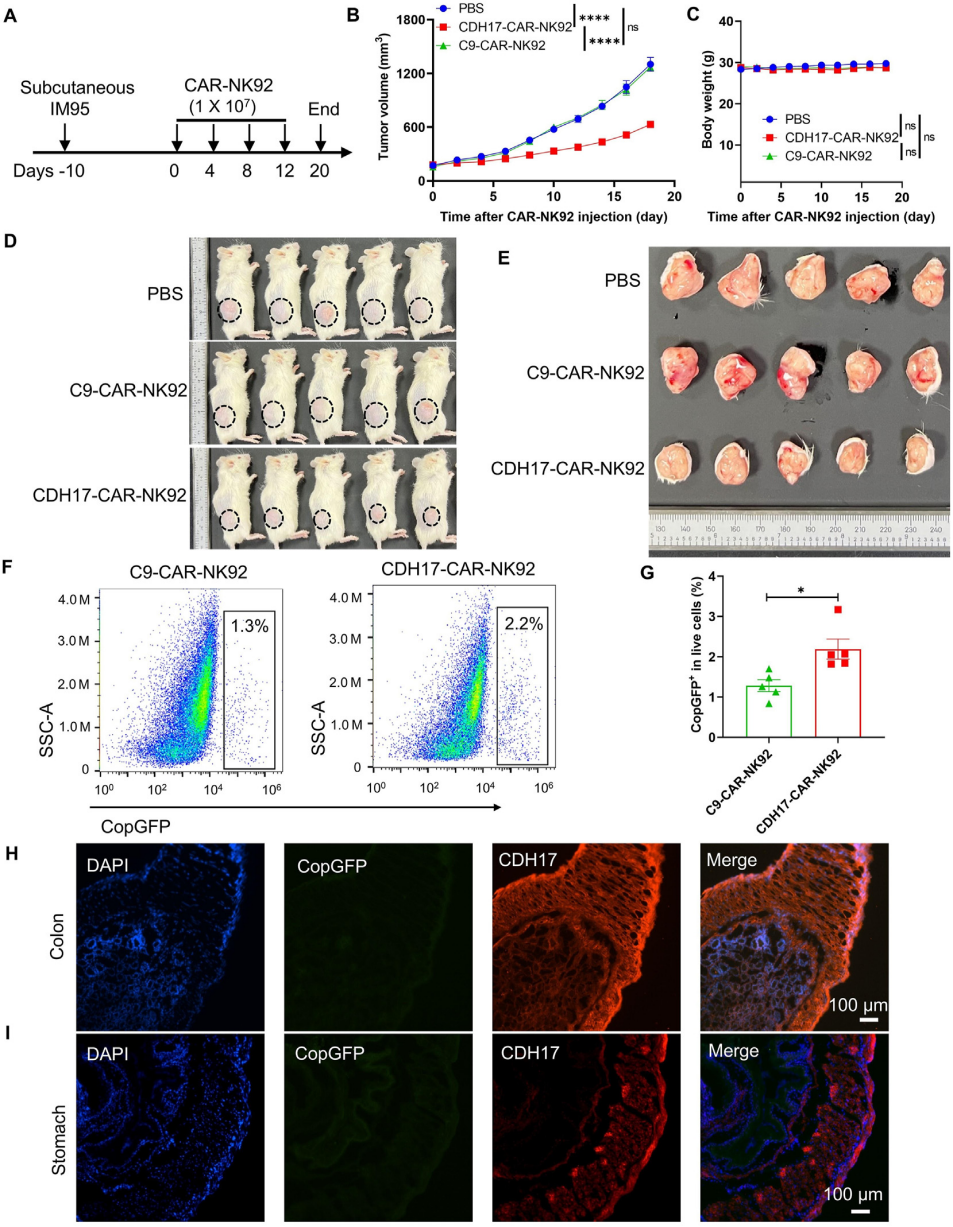
CDH17-targeting CDH17-CAR-NK92 treatment suppresses the progression of IM95 tumor *in vivo*. (A) Diagram of treatment schedule in IM95 xenograft model. NCG mice were subcutaneously injected with IM95 cells on Day −10. Starting from Day 0, CDH17-CAR-NK92 cells or C9-CAR-NK92 (1 × 10^7^ cells) were infused once every 4 days through the tail vein, for a total of 4 injections (*n* = 5). (B) Tumor growth curves with various treatments. (C) Body weight of each group at the indicated time points. (D) Images of tumor-bearing mice from the different treatment groups. (E) Images of excised tumors from the mice with various treatments. (F) The infiltration of CDH17-CAR-NK92 and C9-CAR-NK92 cells in tumors on the fourth day after the final CAR-NK92 administration. CopGFP positive cells were determined by flow cytometry (*n* = 5). (G) Quantification of CopGFP positive cells in the tumors. (H, I) The colon (H) and stomach (I) were stained with anti-CDH17 and anti-CopGFP after CDH17-CAR-NK92 cells treatment. Data are presented as mean ± SEM. The difference for each group was analyzed by two-way ANOVA analysis. ns, no significance; ∗*P* < 0.05, ∗∗∗∗*P* < 0.0001.

To further assess the *in vivo* anti-tumor efficacy of CDH17-CAR-NK92 cells in GC tumors expressing CDH17, IM95 cells were engrafted into NCG mice (Day −10). Starting from Day 0, CAR-NK92 cells (1 × 10^7^ cells) were administered *via* the tail vein every 4 days for a total of 4 injections. This dosing schedule is well supported by a substantial body of prior research (Fig. 3A)^12^^,^^36^, ^37^, ^38^, ^39^. Tumor volume and body weight were monitored every 2 days (Fig. 3B and C). Notably, CDH17-CAR-NK92 cells significantly inhibited tumor growth in mice compared to C9-CAR-NK92 cells (Fig. 3D and E). These results demonstrate that CDH17-CAR-NK92 cells effectively suppress the progression of solid tumors expressing CDH17 on the cell surface. Additionally, the infiltration of CAR-NK92 cells in the tumor was examined using an antibody against CopGFP, which was stably expressed in engineered CAR NK cells. As shown in Fig. 3F and G and Supporting Information Fig. S4, there was an increased presence of CopGFP-expressing cells in tumor tissues treated with CDH17-CAR-NK92 cells compared to the C9-CAR-NK92 cells group. Next, we assessed the infiltration and impact of CDH17-CAR-NK92 cells on the mouse colon and stomach, which typically express CDH17. Our results demonstrated that CDH17-CAR-NK92 cells were not detectable in the colon or stomach of tumor-bearing mice, despite the robust expression of CDH17 in these tissues (Fig. 3H and I). The *in vivo* imaging results with CAR-NK cells labeled with bio-orthogonal reagents azide (–N_3_) and DBCO-ICG^40^^,^^41^ demonstrated that both CDH17-CAR-NK92 and C9-CAR-NK92 cells rapidly reached the tumor site by 24 h post injection, peaking at 48 h post injection (Supporting Information Fig. S5). Strikingly, CDH17-CAR-NK92 cells exhibited markedly higher tumor accumulation at 48 and 72 h, underscoring their targeted localization advantage. However, by 96 and 120 h, while CDH17-CAR-NK92 cells still showed higher accumulation, the difference was no longer significant, highlighting the short persistence of NK cells in tumors and the necessity for multiple administrations to enhance therapeutic efficacy^42^^,^^43^ (Fig. S5B–S5D). *Ex vivo* tumor imaging at 120 h aligned with our *in vivo* observations (Fig. S5E). Although CDH17-CAR-NK92 cells maintained a higher accumulation level, the distinction between the 2 groups had diminished, reinforcing the need for repeated CAR-NK cell transfusions. Additionally, *ex vivo* organ imaging revealed a predominant accumulation of both CAR-NK92 cell types in the tumor, liver, and spleen, with minimal presence in other organs. The non-tumor accumulation of CAR-NK cells is indeed consistent with the natural homing and trafficking patterns of NK cells, which are known to reside in the liver and spleen^42^. These organs play a crucial role in NK cell biology, serving as key sites for their surveillance and immune response^42^^,^^44^. H&E staining further revealed that the heart, liver, spleen, lungs, kidneys, and colon were intact, and no obvious structural damage was observed (Supporting Information Fig. S6). Moreover, there was no significant observed toxicity following CAR-NK92 cell treatment, as indicated by whole blood routine test and serum biochemical test (Supporting Information Figs. S7 and S8), supporting the safety of CDH17 as a therapeutic target. Taken together, these findings suggest that CDH17-targeting CDH17-CAR-NK92 cells can efficiently suppress the progression of GC solid tumors *in vivo*.

### 3.4 CDH17-targeting CAR-NK cells significantly inhibit tumor progression in a patient-derived gastric xenograft (PDX) model

**Figure 4:**
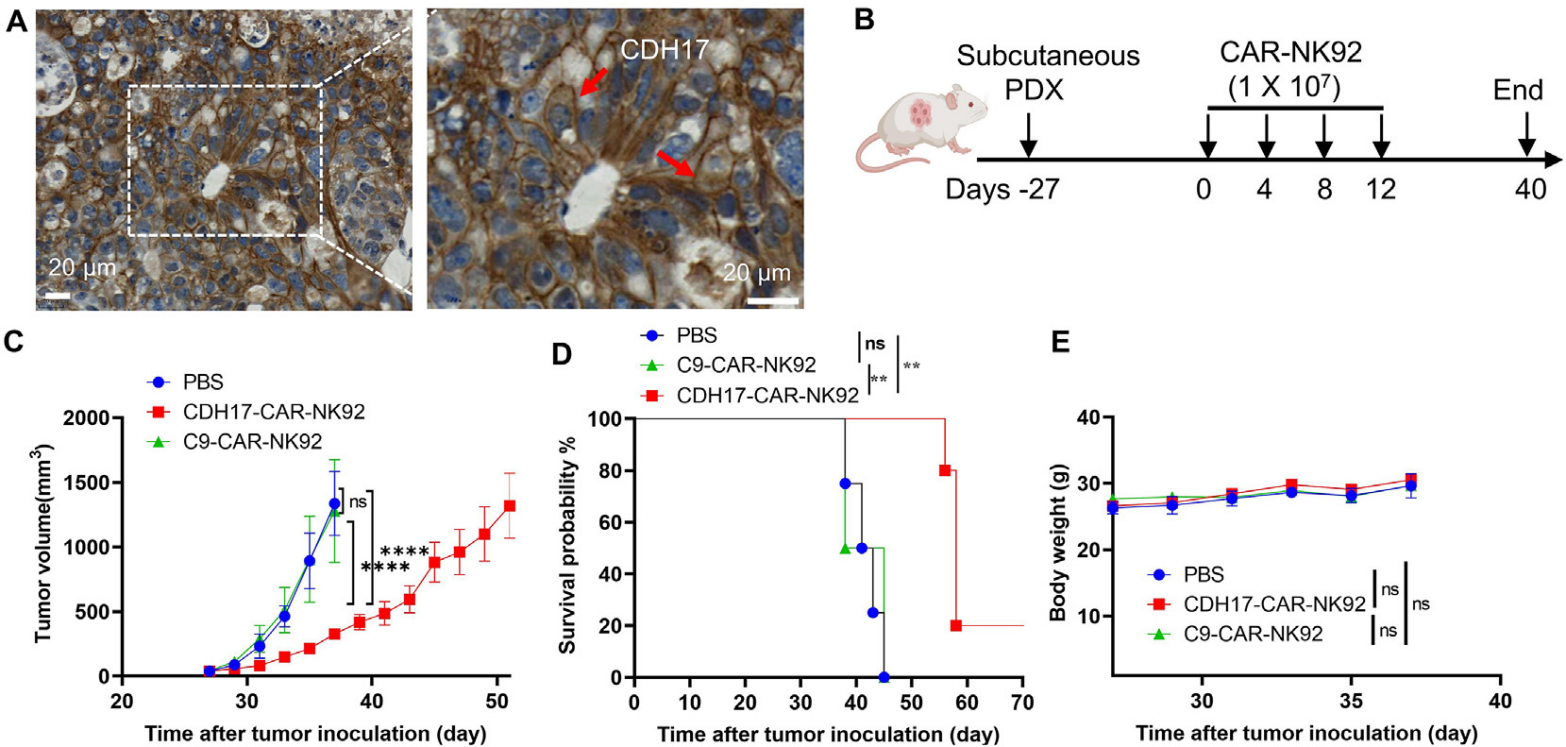
CDH17-targeting CDH17-CAR-NK92 cells control gastric cancer progression in a PDX mouse model. (A) Examination of CDH17 expression in a PDX model. The expression of CDH17 protein in gastric cancer PDX samples was assessed by IHC. Scale bar, 20 μm. (B) Diagram of experimental schedule in the gastric cancer PDX model. NCG mice were subcutaneously implanted with gastric cancer PDX samples on Day −27. When tumor volume reached ∼50 mm^3^, mice were randomly grouped. Starting from Day 0, either CDH17-CAR-NK92 cells or C9-CAR-NK92 cells (1 × 10^7^ cells) were given once every 4 days through the tail vein, for a total of 4 injections. (C) Tumor growth curves in PDX mice (*n* = 4–5). The differences for each group were analyzed by two-way ANOVA analysis. (D) Kaplan–Meier survival curves of tumor-bearing mice after treatment with CAR-NK92 cells. The difference was analyzed by log-rank (Mantel–Cox) test. (E) The body weight of each group was measured at the indicated time points (*n* = 4–5). Data are presented as mean ± SEM. The differences were analyzed by two-way ANOVA analysis. ns, no significance; ∗∗*P* < 0.01, ∗∗∗∗*P* < 0.0001.

Patient-derived xenograft (PDX) models have garnered considerable attention in tumor studies due to their high similarity to human tumors in components and heterogeneity^45^. Given that CDH17-targeting CDH17-CAR-NK92 therapy showed excellent anti-tumor activity in the CDX model, we next investigated whether CDH17-CAR-NK92 therapy could obtain similar results in a PDX model. A gastric cancer tissue-derived PDX model (5th passage) was utilized to assess therapeutic effects. The expression level of CDH17 on tumor tissues from this PDX model was initially determined by IHC, revealing high surface expression of CDH17 in PDX samples (Fig. 4A). *In vivo* anti-tumor effects of CAR-NK92 in the PDX model were then conducted according to the experimental schedule shown in Fig. 4B. NCG mice were subcutaneously implanted with GC PDX samples (Day −27). On Day 0, mice were randomly grouped when the tumor volume reached ∼50 mm^3^. CAR-NK92 cells (1 × 10^7^ cells) were administered once every 4 days *via* the tail vein for a total of 4 injections. The results demonstrated that CDH17-targeting CDH17-CAR-NK92 cells significantly impeded tumor growth and extended mouse survival compared to the C9-CAR-NK92 cells (Fig. 4C and D, Supporting Information Fig. S9). Moreover, there was no noticeable weight loss observed in any of the mice throughout the entire treatment process (Fig. 4E). Overall, these findings indicate that CDH17-targeting CAR-NK cells hold significant potential for clinical translation.

### 3.5 CDH17-targeting CAR-NK cells effectively suppress refractory PC tumor *in vivo*

**Figure 5:**
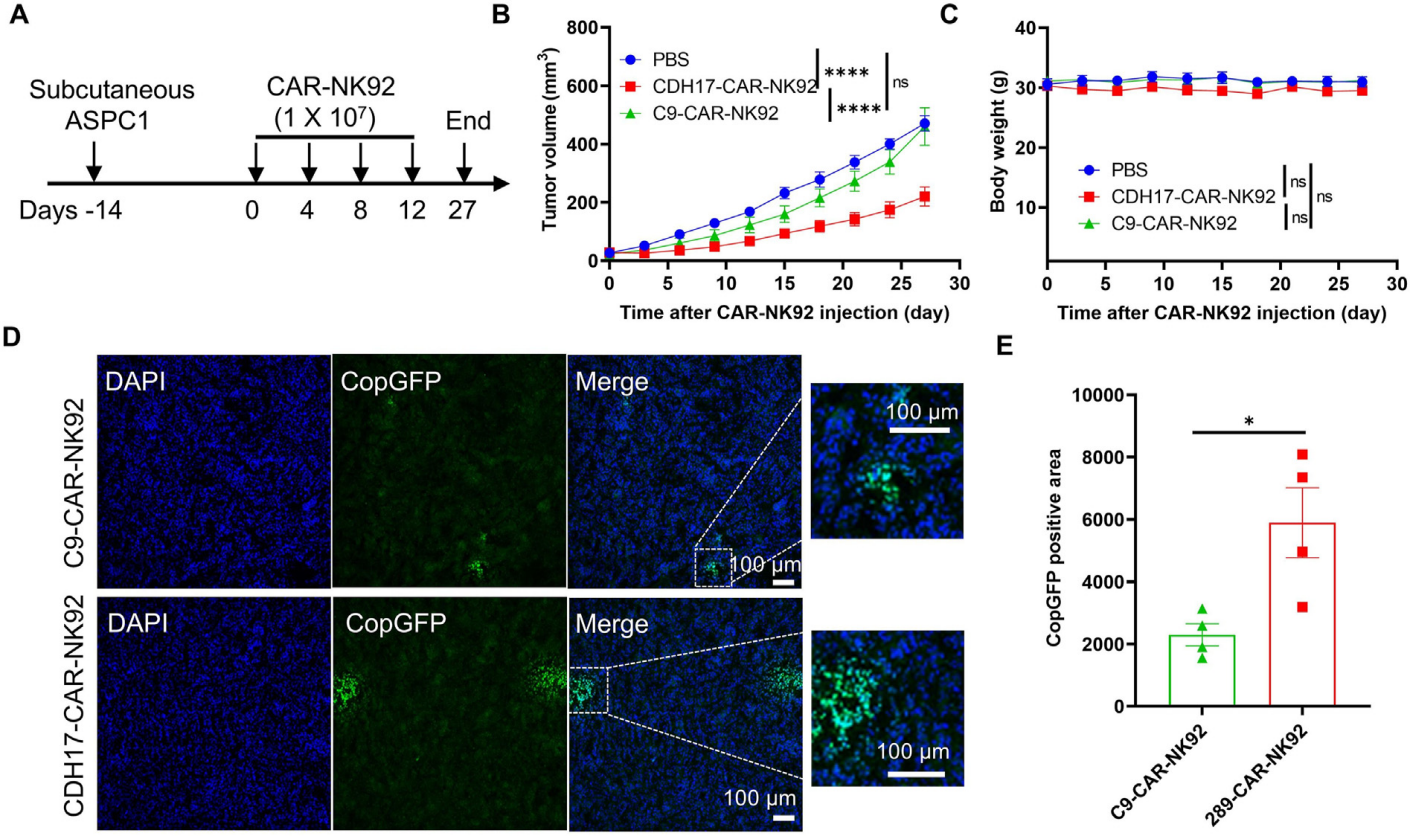
CDH17-targeting CDH17-CAR-NK92 cells retard PC progression in ASPC1 mouse model. (A) Diagram of experimental schedule in the pancreatic cancer ASPC1 model. NCG mice were subcutaneously implanted with ASPC1 cells on Day −14. When tumor volume reached ∼50 mm^3^, mice were randomly grouped. Starting from Day 0, either CDH17-CAR- NK92 cells or C9-CAR-NK92 cells (1 × 10^7^ cells) were given once every 4 days through the tail vein, for a total of 4 injections. (B) Tumor growth curves in ASPC1 tumor-bearing mice (*n* = 6). Data are presented as mean ± SEM. The differences for each group were analyzed by two-way ANOVA analysis. (C) The body weight of each group was measured at the indicated time points. Data are presented as mean ± SEM. The differences were analyzed by two-way ANOVA analysis. (D) The infiltration of CDH17-CAR-NK92 and C9-CAR- NK92 cells in tumors after treatment. CopGFP positive cells were determined by immunofluorescence staining with anti-CopGFP antibody. Scale bar 100 μm. (E) Quantification of CopGFP-positive area in the tumors. Data are presented as mean ± SEM. *P* values were calculated based on the unpaired Student's *t*-test. ns, no significance; ∗*P* < 0.05, ∗∗∗∗*P* < 0.0001.

PC presents a clinical challenge due to the absence of symptoms in early-stage disease and a unique tumor microenvironment characterized by dense desmoplasia, high infiltration of fibroblasts, and immunosuppressive cells^46^. Investigating novel therapeutic approaches for the treatment of PC holds great significance. To assess the efficacy of CDH17-CAR-NK92 cells in expressing CDH17 PC tumors *in vivo*, CDH17-positive ASPC1 cells were engrafted into NCG mice (Day −14). Starting from Day 0, CAR- NK92 cells (1 × 10^7^ cells) were administered *via* the tail vein every 4 days for a total of 4 injections (Fig. 5A). Tumor volume and body weight were monitored every 2 days (Fig. 5B and C). Surprisingly, CDH17-CAR-NK92 cells significantly inhibited ASPC1 tumor burden in mice compared to C9-CAR-NK92 cells, which did not exhibit any tumor inhibition (Fig. 5B, Supporting Information Fig. S10). These data illustrate that CDH17-CAR-NK92 cells can also suppress the growth of CDH17-positive PC solid tumors. No significant toxicity was observed after CAR-NK92 cell treatment, as evidenced by stable body weight, whole blood routine test, serum biochemical test, and histological analysis of vital organs (Fig. 5C, Supporting Information Figs. S11 and S12). Finally, the infiltration of CAR-NK92 cells in the tumor was analyzed. As shown in Fig. 5D and E, the number of cells expressing CopGFP was increased in tumor tissues treated with CDH17-CAR-NK92 cells compared to the C9-CAR-NK92 cell groups. Taken together, these results indicate that CDH17-targeted CDH17-CAR-NK92 cells are also effective in impeding the progression of formidable PC solid tumors *in vivo*.

### 3.6 Blocking CD47 enhances the antitumor effects of CDH17-targeting CAR-NK cells against the PC mouse model

**Figure 6:**
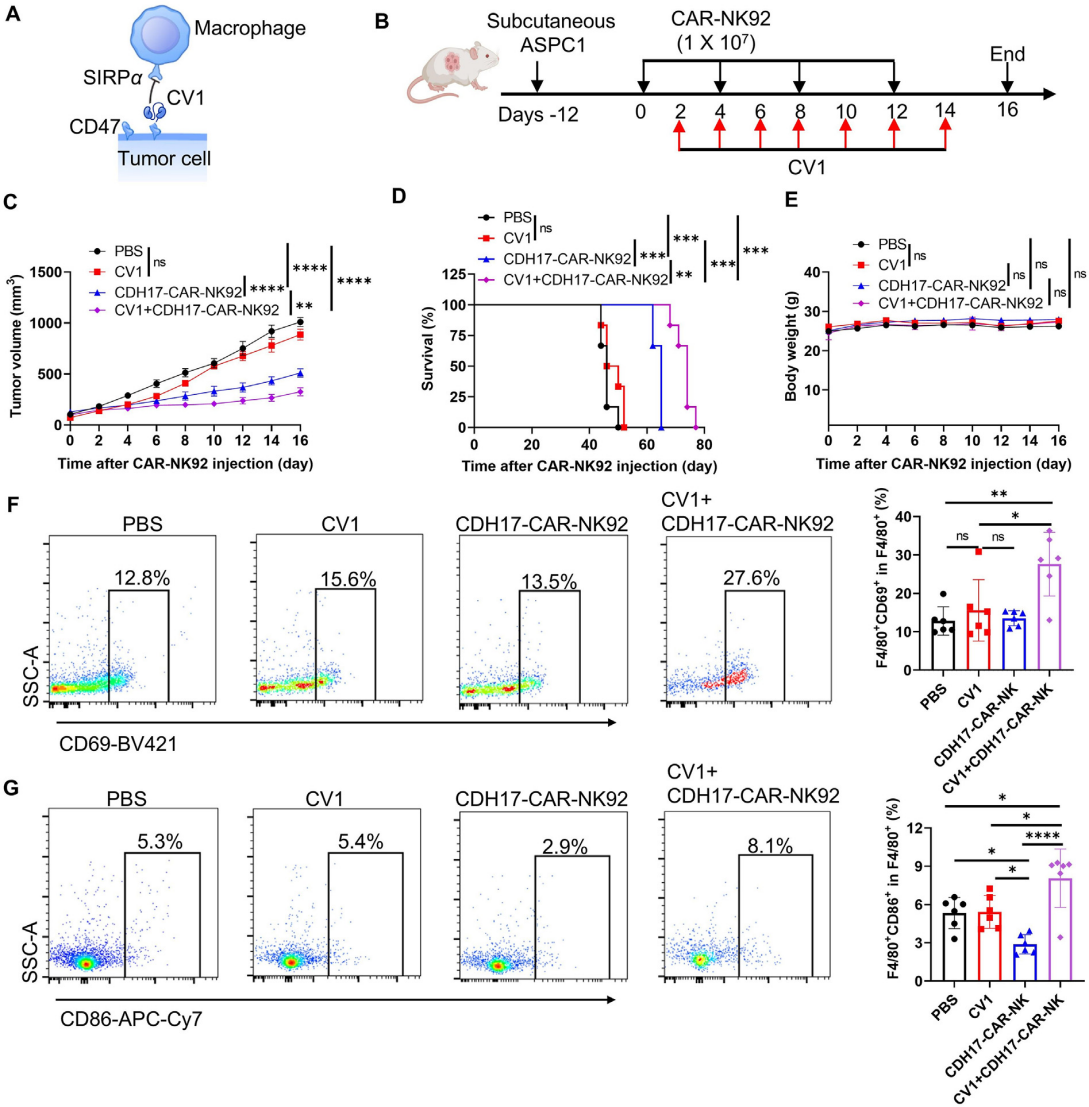
Combination of CDH17-targeting CDH17-CAR-NK92 cells therapy with CD47 blocker synergistically represses pancreatic cancer (PC) progression in the ASPC1 mouse model. (A) Schematic diagram of the CV1 blocking CD47–SIRP*α* signaling axis. (B) Diagram of experimental schedule in the ASPC1 pancreatic cancer model. (C) Tumor growth curves in ASPC1 mice (*n* = 6). All the data are presented as mean ± SEM. The differences for each group were analyzed by two-way ANOVA analysis. (D) Kaplan–Meier survival curves of ASPC1 tumor-bearing mice after treatment. The *P* value was analyzed by log-rank (Mantel–Cox) test. (E) The body weight of each group was measured at the indicated time points. Data are presented as mean ± SEM. The differences were analyzed by two-way ANOVA analysis. (F) Representative flow cytometric analysis of the number of macrophage activation (CD45^+^Gr1^–^F4/80^+^CD69^+^) in tumor. Data are presented as mean ± SEM. The differences were analyzed by one-way ANOVA analysis. (G) Representative flow cytometric analysis of the number of M1-phenotype macrophages (CD45^+^Gr1^–^F4/80^+^CD86^+^) in the tumor. Data are presented as mean ± SEM. The differences were analyzed by one-way ANOVA analysis. ns, no significance; ∗*P* < 0.05, ∗∗*P* < 0.01, ∗∗∗*P* < 0.001, ∗∗∗∗*P* < 0.0001.

Inspired by the facts that disrupting the CD47-SIRP*α* signaling axis with CV1 enhances the anti-tumor efficacy of CAR-T cells^25^, and that CD47 blockades can improve the activation and recruitment of NK cells^47^, we further wonder whether CV1 could augment the effectiveness of CDH17-CAR-NK92 cells (Fig. 6A). NCG mice were subcutaneous injected with ASPC1 cells at Day −14 (Fig. 6B). Starting from Day 0, CAR- NK92 cells (1 × 10^7^ cells) were given *via* tail vein every 4 days for a total of 4 injections (Fig. 6B). Starting from Day 2, CV1 was administered *via* intraperitoneal injection at 200 μg every 2 Days for a total of 7 injections (Supporting Information Fig. S13). The tumor volume and body weight were monitored every 2 days (Fig. 6C, Supporting Information Fig. S14). No significant toxicity was observed after CV1 treatment alone or in combination with CAR-NK92 cells based on body weight loss and histological analysis of vital organs (Fig. 6E, Supporting Information Fig. S15). While CV1 alone did not markedly reduce tumor size compared to the PBS control, its addition significantly enhanced the anti-tumor effects and improved mouse survival of CDH17-CAR-NK92 cells compared with CDH17-CAR-NK92 or CV1 monotherapy (Fig. 6C and D). To explore the possible mechanism by which CV1 promotes the anti-tumor effect of CDH17-CAR-NK92 cells, we performed flow cytometry analysis for macrophages in the tumor microenvironment after treatment (Fig. 6F and G, Supporting Information Fig. S16). The results demonstrated a significant increase in the number of activated macrophages (CD45^+^Gr1^–^F4/80^+^CD69^+^) in the group treated with a combination of CV1 and CDH17-CAR-NK92 cells (Fig. 6F). In contrast, the groups treated with either CV1 or CDH17-CAR-NK92 cells alone showed a slight, though not statistically significant, elevation in the number of CD69^+^ macrophages compared to the PBS group (Fig. 6F). The CV1 monotherapy group showed no impact on M1-phenotype macrophages (CD45^+^Gr1^–^F4/80^+^CD86^+^) compared to the PBS group (Fig. 6G). Additionally, CV1 had no significant effect on the number of CDH17-CAR-NK92 cells in the tumors (Supporting Information Fig. S17). Surprisingly, the group treated with CDH17-CAR-NK92 cells alone exhibited a significant reduction in the number of M1-phenotype macrophages compared to the PBS group (Fig. 6G). Nevertheless, CV1 treatment was able to effectively reverse the decrease in the number of M1-phenotype macrophages caused by the CDH17-CAR-NK92 cells treatment, and this ameliorative effect was significant even when compared with the CV1 monotherapy group (Fig. 6G). Taken together, these findings suggest that CV1 may enhance the anti-tumor effect of CDH17-CAR-NK92 cells by increasing the number of M1-phenotype macrophages and enhancing overall macrophage activation in the tumors.

## 4 Discussion

Gastrointestinal (GI) cancers, encompassing malignant neoplasms in the GI tract and digestive organs, represent a leading cause of cancer-related mortality worldwide^48^. Despite advances in treatment, the overall survival rates remain unsatisfactory, and new therapeutics are needed. Here, we demonstrate that CDH17 is expressed on the cell surface of multiple GI cancers, making CDH17 an attractive target for CAR-NK cell therapy. Our results indicate a predominant localization of CDH17 on the lateral side rather than the luminal or basal side of the colon. This unique distribution may confer protection to the colon from circulating CAR-NK cells, which exhibit limited penetration through tight junctions. Our investigation demonstrates the potent anti-tumor activity of CDH17-CAR-NK cells against CDX and PDX mouse models. We also disclose that CV1 could significantly enhance the anti-tumor effect of CDH17-CAR-NK92 cells, with one plausible mechanism being the enhancement of macrophage activation.

Adoptive immune cell transfer (ACT) has emerged as a promising approach in cancer immunotherapy, involving the use of genetically modified immune cells such as T cells and NK cells. Among these, CAR-T cell therapy has been a notable success, gaining clinical application across various cancer types^49^, ^50^, ^51^. However, CAR-T therapy still has a few limitations, including the risk of graft-*versus*-host disease (GVHD), cytokine release syndrome (CRS), immune effector cell-associated neurotoxicity syndrome (ICANS), and the high associated production costs^52^^,^^53^. Furthermore, the isolation and utilization of autologous T cells present substantial challenges in this approach. To address these limitations, exploring alternative tumoricidal agents may provide a solution to the aforementioned drawbacks.

In contrast to T cell therapy, NK cell-based ACT has shown no association with CRS or ICANS, irrespective of the use of autologous or allogeneic NK cells^9^^,^^54^. One possible reason for this outcome could be that NK cells do not proliferate and secrete cytokines as efficiently as T cells *in vivo*^10^. Furthermore, the diverse sources of NK cells, including induced pluripotent stem cells, umbilical cord blood, peripheral blood, and various NK cell lines, contribute to the feasibility of developing and clinically utilizing CAR-NK cells^9^. Thus, NK cells might be superior to T cells in this context. Numerous studies have explored the application of CAR-NK cells in treating solid tumors, confirming the potential effectiveness and safety of CAR-modified NK-92 cells as immunotherapy for solid malignancies^12^^,^^13^^,^^55^. In this study, we evaluated the partial safety of CDH17-CAR-NK92 cells by examining parameters such as body weight loss, histological analysis, whole blood routine, and serum biochemical tests in the tail vein blood of NOD/SCID mice. Notably, no significant toxicity was observed based on these evaluations. Furthermore, although substantial numbers of CDH17-CAR-NK92 cells successfully infiltrated into tumors, no such infiltration was observed in the stomach and colon, where CDH17 is highly expressed. This may be related to the expression pattern of CDH17. We have shown that CDH17 is predominantly localized on the lateral side of the colonic epithelium, in contrast to the luminal or basal sides (Fig. 1J and K). Conversely, in the subcutaneous tumors of mice bearing ASPC1 cells, CDH17 expression is notably distributed around the tumor cells (Fig. 1L and M). This distinct distribution likely restricts the access of circulating CDH17-CAR-NK cells to normal colon tissue, as it limits their contact with CDH17 proteins on healthy cells, which is consistent with a previous study^34^. Furthermore, our *in vivo* imaging data (Fig. S5) demonstrate that both CDH17-CAR-NK and C9-CAR-NK cells primarily accumulate in the liver, spleen, and tumors, with no significant accumulation observed in CDH17-expressing organs such as the colon and stomach (Fig. S5E). This selective targeting is further supported by the fact that CAR-NK cells do not accumulate in CDH17-expressing organs where the pattern of CDH17 expression is different from tumor cells. Furthermore, NK92 cells have already been demonstrated to be a safe and effective antitumor therapy and have received FDA approval^56^. Hence, the use of CDH17-targeted CAR-NK92 cells appears to be a safe therapeutic approach to treat GI cancers.

A crucial issue of CAR-NK cell therapy is the establishment of stable CAR expression on the surface of NK cells. Currently, single-chain variable fragment (scFv) is widely used for CAR construction^57^. In fact, scFv-based CAR has some unavoidable drawbacks. The deletion of the constant region in scFv may lead to auto-aggregation due to high hydrophobicity and loss of targeting^58^. Furthermore, CAR aggregation could result in excessive cytotoxic signaling independent of tumor antigens, potentially inducing premature T cell anergy^59^. In light of these considerations, we opted to employ nanobodies (Nbs) for CAR construction in this study. Nbs are distinctive antibody fragments derived from heavy-chain antibodies discovered in camelids and chondrichthyes, consisting of only 2 heavy chains^60^. This unique structure equips Nbs with several advantages over scFv when serving as CAR molecules. The longer CDR3 sequence in Nbs provides flexibility in conformational expansion, enabling them to reach epitopes typically inaccessible to conventional antibodies^61^. Additionally, the gene sequence of Nbs exhibits high homology with the human VH gene family III sequence, resulting in low immunogenicity and strong compatibility with humans^62^. Finally, Nbs rarely display auto-aggregation due to high hydrophobicity, a common phenomenon observed with scFv^63^. Previously, our group screened for CDH17 targeting Nbs using a VHH phage display library comprising ∼2 × 10^9^ independent clones and found that Nb CDH17 had a high binding affinity to both human and mouse CDH17. The ability of Nb CDH17 to bind to both human and mouse CDH17 allowed for a more comprehensive evaluation of its safety. Consequently, Nb CDH17 was selected for the construction of CDH17-targeting CAR-NK cells. Following numerous generations of culturing, the surface expression of the CAR on CAR-NK92 cells remained unchanged and maintained the same killing ability, indicating that the CAR molecule constructed by Nb CDH17 was stable (data not shown).

Despite the remarkable success of CAR-NK cell therapy, the immunosuppressive microenvironment remains a barrier to therapeutic efficacy. To overcome this, the integration of CAR-NK cells with immune checkpoint inhibitors is under investigation in numerous studies, indicating better therapeutic effects compared with single therapy in clinical applications^64^^,^^65^. Therefore, in this study, we aimed to combine a checkpoint inhibitor against the CD47–SIRP*α* axis that has not been previously utilized in combination with CAR-NK therapy to enhance the anti-tumor efficacy of CDH17-targeted CAR-NK cells. CD47 blockade in various contexts of tumor therapies has shown promising outcomes but with complicated mechanisms involving the interaction of different immune cells in TME, such as NK cells, T cells, and even DCs^47^^,^^66^^,^^67^. A previous study demonstrated that the combination of CAR-T cell therapy with the CD47–SIRP*α* axis blocker CV1 can potentiate anti-tumor effects^25^. However, the potential synergistic effects of CV1 with CAR-NK therapy have not been explored to date. Therefore, this study conducted an assessment of the combined therapeutic effects of CDH17-targeted CAR-NK with CV1. As expected, CV1 significantly improved the anti-tumor effects of CDH17-CAR-NK92 cells against the ASPC1 tumor model. Although CV1 has shown some antitumor effects in certain studies^23^^,^^68^, other research has demonstrated that CV1 monotherapy does not yield significant antitumor outcomes^26^, and it is essential to combine it with other treatments such as therapeutic antibodies, chemotherapy, or CAR-T therapy^23^^,^^24^^,^^26^^,^^69^, which is consistent with our findings. Additionally, pancreatic cancer is notoriously resistant to a wide range of treatments. This discrepancy about CV1 monotherapy may be due to differences in tumor models and treatment schedules. Further investigations revealed that CV1 can stimulate the activation of macrophages in the tumor microenvironment, leading to an increase in M1-phenotype macrophages. This finding is consistent with previous results that anti-CD47 treatment can induce the transformation of M2-like TAMs into the M1-phenotype macrophages^70^, and reprogramming macrophage differentiation into M1-phenotype can enhance the effectiveness of T cell immunotherapy^71^. Specifically, reactivating TAMs and MDSCs using a folate-targeted Toll-like receptor 7 agonist (FA-TLR7-1A) can also markedly enhance the effectiveness of CAR T cell therapy^72^. Additionally, blockade of this pathway with CV1 and other anti-CD47 agents has been demonstrated to improve macrophage and dendritic cell (DC) cross-priming of CD8^+^ T cells for antitumor responses^23^^,^^66^^,^^68^^,^^73^. Our study also found that treatment with CV1 in combination with CDH17-CAR-NK92 led to an increase in CD69^+^ and CD86^+^ macrophages, potently implicating the enhanced antitumor activity of tumor-associated macrophages. The precise mechanisms driving this enhancement require further exploration, particularly using immunocompetent mice, to fully understand the interplay between CAR-NK cells, macrophages, and other immune cells in the tumor microenvironment.

Our studies have indicated a modest reduction in F4/80 CD86 macrophages following treatment with CDH17-CAR-NK cells. However, the precise mechanism behind this phenomenon remains elusive, and we acknowledge this as a limitation in our current study. The complex interplay between CAR-NK cells and macrophages in the tumor microenvironment (TME) has been a subject of increasing interest. Recent studies have shed light on several potential mechanisms, which might explain the observed reduction in F4/80^+^CD86^+^ macrophages, including but not limited to CAR-NK secreted cytokine-mediated effects^74^, competition for survival factors for both NK cells and macrophages^75^^,^^76^, direct cytotoxicity of CAR-NK cells to TAM^77^, alteration of CAR-NK-induced chemokine profiles in TME^78^, etc. Our findings, contextualized within recent literature, suggest that the interplay between CAR-NK cells and macrophages in the TME is complex and multifaceted. The modest reduction in F4/80^+^CD86^+^ macrophages observed with CDH17-CAR-NK cell treatment may result from a combination of the mechanisms described above. The enhanced efficacy observed with the addition of CD47 blockade (CV1) aligns with recent findings in the field^23^^,^^26^. CD47 acts as a “don't eat me” signal, and its blockade enhances macrophage activation (CD69^+^) and phagocytosis against tumor cells^79^. The increase in F4/80^+^CD86^+^ and F4/80^+^CD69^+^ macrophages with CV1 treatment may represent an influx of newly recruited and activated macrophages or a repolarization of existing macrophages towards a more anti-tumoral phenotype. Furthermore, these observations underscore the importance of considering the entire immune landscape when developing cellular immunotherapies. The synergistic effect observed with the combination of CAR-NK cells and CD47 blockade highlights the potential of targeting multiple aspects of the TME to enhance therapeutic efficacy. Thus, the combination of CAR-NK therapy with TME modulators such as antibodies against PD-1, CTLA4, or CD47, and agonists to the STING pathway or Toll-like receptors (TLR7/8/9)^80^^,^^81^, may be an effective strategy to enhance the antitumor performance of CAR-NK therapy. This approach aligns with emerging strategies in the field that aim to engineer more comprehensive anti-tumor responses by modulating both adaptive and innate immune components. For our study, to gain a deeper understanding of how CDH17-CAR-NK cells reduce F4/80^+^ CD86^+^ macrophages and how CD47 blockade (CV1) enhances the therapeutic effect of CDH17-CAR-NK cells, further studies using immunocompetent or humanized mice are warranted. These studies will provide a more comprehensive view of the complex interactions between CAR-NK cells, macrophages, and other immune components in the TME, leading to more effective therapeutic strategies. In summary, these findings indicate that strategies aimed at activating tumor-associated myeloid cells could significantly augment the potencies of adoptive cell therapies.

## 5 Conclusions

In conclusion, CDH17-targeting CAR-NK92 cells exhibit robust anti-tumor activity in multiple GI cancers, as demonstrated in both CDX and PDX models. Furthermore, our findings highlight the potential of combining CDH17-targeting CAR-NK92 cells with CD47 blocker CV1 to augment the anti-tumor effects. Of note, we have to acknowledge that deep exploration is needed to decipher the detailed mechanisms of how CD47 blockade influences the behaviors of CAR NK cells in TME, and syngeneic tumor models in component or humanized mice are possibly better to analyze the interaction networks of various immune cells after the administration of CAR NK cells. Overall, our work demonstrates that CDH17-targeting CAR-NK92 cells is a safe and effective therapeutic approach to treat GI cancers. Our research also offers an alternative option for the combination therapy with CAR-NK cells.

## Author contributions

Shanchao Zhao, Rui Hou, Wei Zhang, Zhijie Li and Jigang Wang designed research and revised the manuscript. Liuhai Zheng, Youbing Ding, Xiaolong Xu, Huifang Wang, Guangwei Shi, Yang Li, Yuanqiao He, Yue Gong, Xiaodong Zhang, Jinxi Wei, Zhiyu Dong and Jiexuan Li performed experiments. Liuhai Zheng, Youbing Ding, Xiaolong Xu and Huifang Wang analyzed data. Liuhai Zheng, Youbing Ding and Xiaolong Xu wrote the paper. All authors read and approved the final manuscript.

## Conflicts of interest

The authors declare no competing interests.

## Appendix A Supplementary data

The following is the Supporting Information to this article.
