# Supplementary material for: CDH17-targeting CAR-NK cells synergize with CD47 blockade for potent suppression of gastrointestinal cancers
Source: Acta Pharm Sin B. 2025 Mar 19;15(5):2559–74. doi: 10.1016/j.apsb.2025.03.039 (PMC12144966; doi:10.1016/j.apsb.2025.03.039)
Supplement: Multimedia component 1 [file mmc1.pdf]

Supporting Information for

Original article

CDH17-targeting CAR-NK cells synergize with CD47 blockade for potent suppression of gastrointestinal cancers

Liuhai Zheng[a,b,†], Youbing Ding[a,c,†], Xiaolong Xu[a,b,†], Huifang Wang[a,b,†], Guangwei Shi[d], Yang Li[a], Yuanqiao He[e,f], Yue Gong[a], Xiaodong Zhang[c], Jinxi Wei[a], Zhiyu Dong[a], Jiexuan Li[a], Shanchao Zhao[g,h,i,*], Rui Hou[a,*], Wei Zhang[a,j,*], Jigang Wang[a,j,k,l,m,n,*], Zhijie Li[a,*]

[a]*Department of Critical Care Medicine, Guangdong Provincial Clinical Research Center for Geriatrics, Shenzhen Clinical Research Centre for Geriatrics, Department of Nuclear Medicine, Shenzhen People's Hospital (the First Affiliated Hospital, Southern University of Science and Technology, the Second Clinical Medical College, Jinan University), Shenzhen 518020, China*

[b]*Integrated Chinese and Western Medicine Postdoctoral Research Station, Jinan University, Guangzhou 510632, China*

[c]*Department of Medical Imaging, the Third Affiliated Hospital of Southern Medical University (Academy of Orthopedics Guangdong Province), Guangzhou 510630, China*

[d]*Department of Neurosurgery & Medical Research Center, Shunde Hospital, Southern Medical University (the First People's Hospital of Shunde Foshan), Guangzhou 510515, China*

[e]*Center of Laboratory Animal Science, Nanchang University, Nanchang 330031, China*

[f]*Key Laboratory of New Drug Evaluation and Transformation of Jiangxi Province Nanchang 330031, China*

[g]*Department of Urology, the Fifth Affiliated Hospital, Southern Medical University, Guangzhou 510900, China*

[h]*Department of Urology, the Third Affiliated Hospital of Southern Medical University, Guangzhou 510630, China*

[i]*Department of Urology, Nanfang Hospital, Southern Medical University, Guangzhou 510515, China*

[j]*Center for Drug Research and Development, Guangdong Provincial Key Laboratory for Research and Evaluation of Pharmaceutical Preparations, Guangdong Pharmaceutical University, Guangzhou 510006, China*

[k]*State Key Laboratory for Quality Ensurance and Sustainable Use of Dao-di Herbs,*

Artemisinin Research Center, Institute of Chinese Materia Medica, China Academy of Chinese Medical Sciences, Beijing 100700, China

[l]*Department of Oncology, the Affiliated Hospital of Southwest Medical University, Luzhou 646000, China*

[m]*Department of Traditional Chinese Medicine and School of Pharmaceutical Sciences, Southern Medical University 510515, China*

[n]*State Key Laboratory of Antiviral Drugs, School of Pharmacy, Henan University, Kaifeng 475004, China*

Received 16 May 2024; received in revised form 13 November 2024; accepted 20 December 2024

[*]Corresponding authors.

E-mail addresses: li.zhijie@szhospital.com (Zhijie Li), jgwang@icmm.ac.cn (Jigang Wang), rui.hou@uwa.edu.au (Rui Hou), lulululu@smu.edu.cn (Shanchao Zhao), zhwei513@connect.hku.hk (Wei Zhang).

[†]These authors made equal contributions to this work.

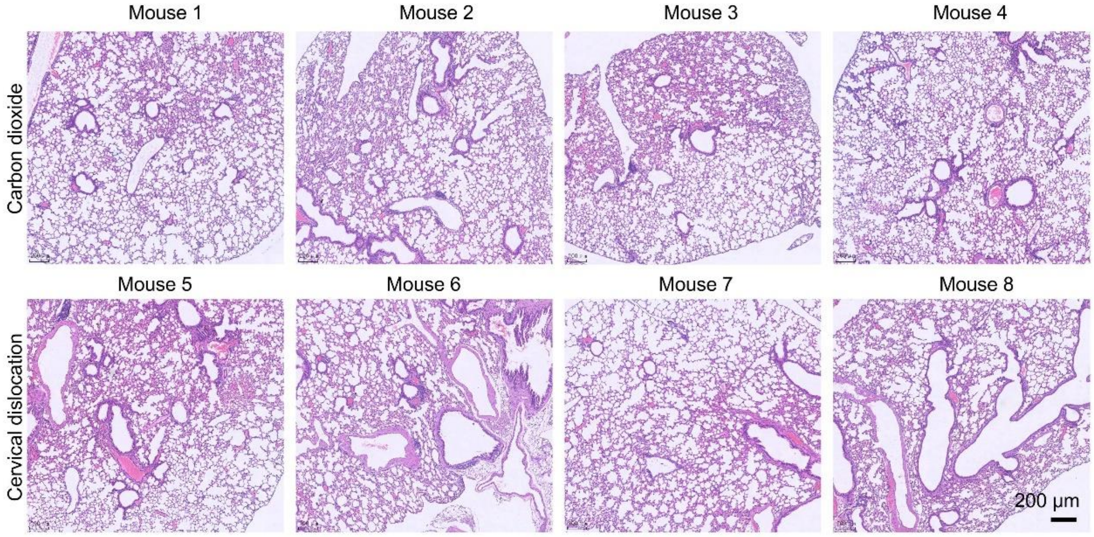

Figure S1 H&E staining of mouse lung tissues from different euthanasia methods. Eight 10-week-old BALB/c mice were randomly assigned to two euthanasia groups (*n* = 4 per group): (1) carbon dioxide euthanasia and (2) isoflurane anesthesia followed by cervical dislocation. In the carbon dioxide euthanasia group, gas flow was initiated at a rate of 1.2 L/min (30% of the chamber's volume per minute) and gradually increased to 2.8 L/min (70% of the chamber's volume per minute). After the mice lost consciousness, carbon dioxide exposure was continued for an additional two minutes to ensure euthanasia before lung tissue collection for histological analysis. In the isoflurane anesthesia group, mice were deeply anesthetized with isoflurane before euthanasia by cervical dislocation, after which lung tissues were collected for H&E staining. Scale bar: 200 μm.

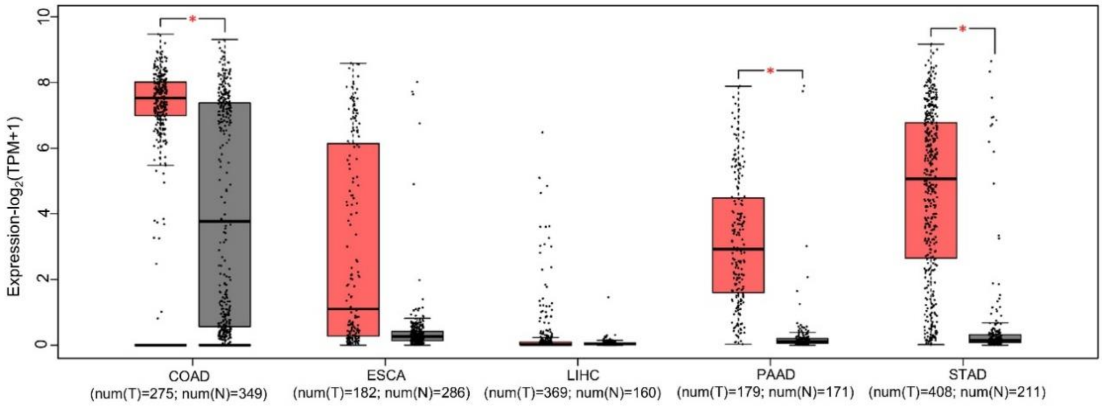

Figure S2 The dot plot illustrating cadherin 17 (*Cdh17*) gene expression levels across various tumor types, generated by gene expression profiling interactive analysis 2 (GEPIA2) website. Normal data include the cancer genome atlas (TCGA) normal tissues and the genotype-tissue expression (GTEx) data. Differential gene expression was conducted using the LIMMA method. The thresholds for $\log_2$ fold change ($|Log_2FC|$) and *q*-value were set at 1 and 0.01, respectively. The difference was analyzed by Student's *t*-test. $^*P < 0.05$.

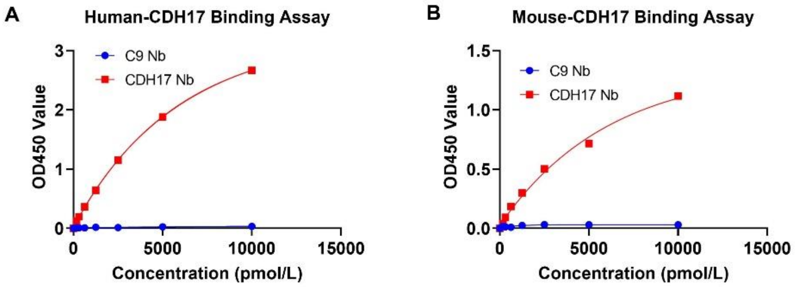

Figure S3 ELISA assessment for CDH17 nanobody (Nb). The human (A) and mouse CDH17 protein (B) in PBS were immobilized on ELISA plates at a concentration of 10 μg/mL overnight at 4 ℃. Serial dilutions of HA-tagged CDH17 Nb or an irrelevant Nb control (C9) in 0.1% PBST were incubated with the immobilized antigen, followed by incubation with HRP-coupled anti-HA.

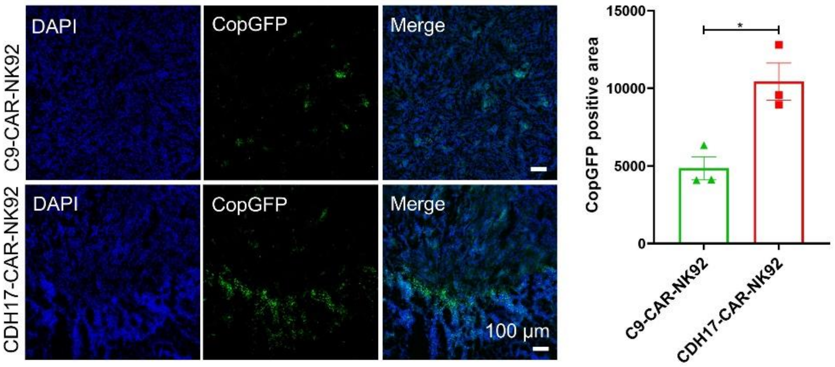

Figure S4 The infiltration of CDH17-CAR-NK92 and C9-CAR- NK92 cells in tumors after treatment. CopGFP positive cells were determined by immunofluorescence staining with anti-CopGFP antibody (n = 3). Quantification of CopGFP-positive area in the tumors. The difference was analyzed by Student's t-test. Data are presented as mean ± SEM. $^{*}P < 0.05$. Scale bar 100 μm.

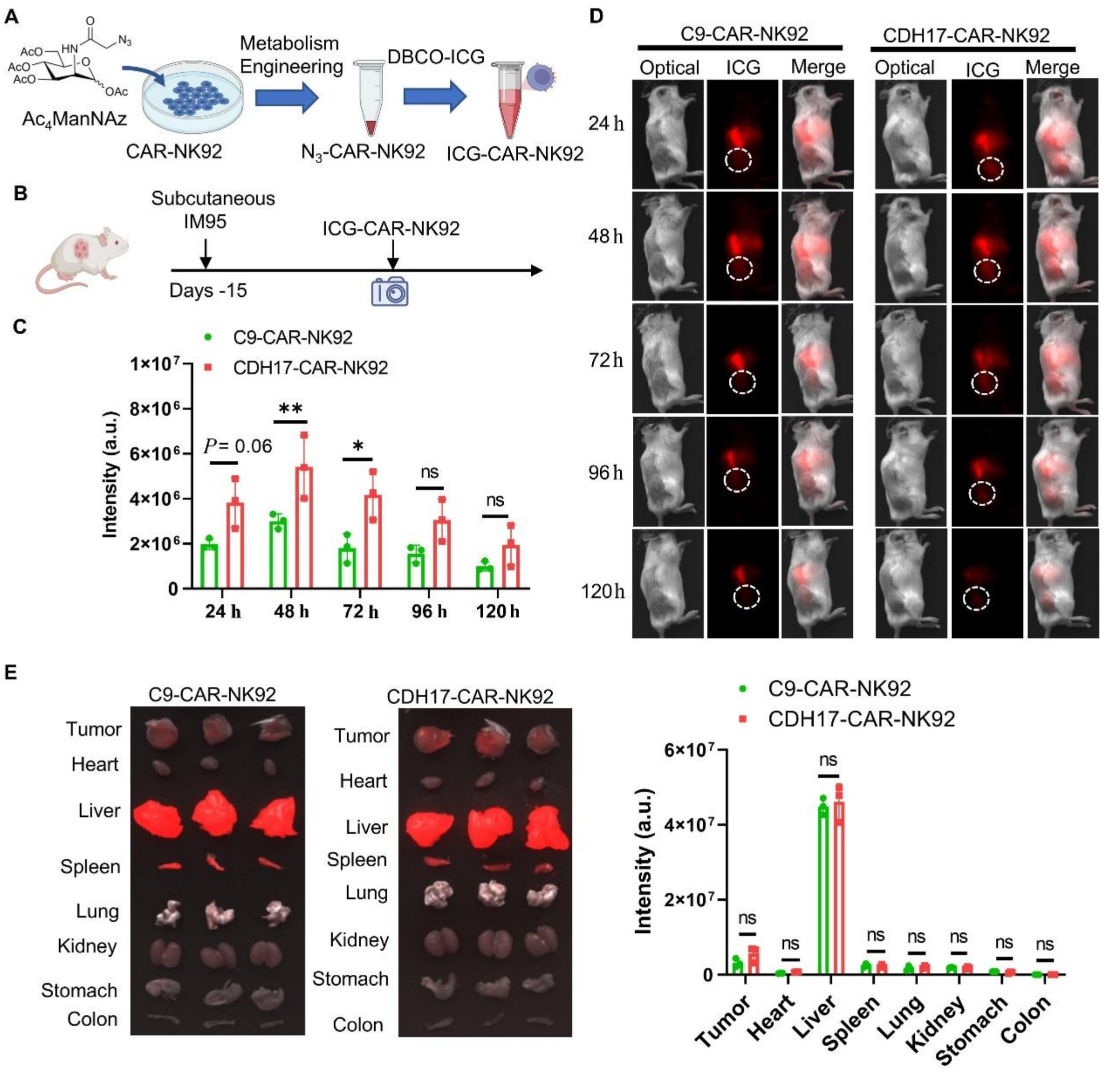

Figure S5 Biodistribution and tumor targeting of CDH17 CAR-NK cells in IM95

tumor-bearing mice. (A) Bio-orthogonal reagents, azide (–N_3), and DBCO-ICG were introduced to the surface of CAR-NK cells through metabolic engineering. (B) Treatment schedule for subcutaneous IM95 tumors using ICG-coupled CAR-NK cells. (C) Quantification of CAR-NK cell accumulation in tumors at different time intervals (n = 3). The difference was analyzed by Student's t-test (D) *In vivo* optical imaging of IM95 tumor-bearing mice at various time points after intravenous injection of ICG-labeled CAR-NK cells. (E) *Ex vivo* imaging analysis of organs harvested from mice 120 hours post-injection (n = 3). The difference was analyzed by Student's t-test. Data are presented as mean ± SEM. ns, no significance; $^{*}P < 0.05$, $^{**}P < 0.01$.

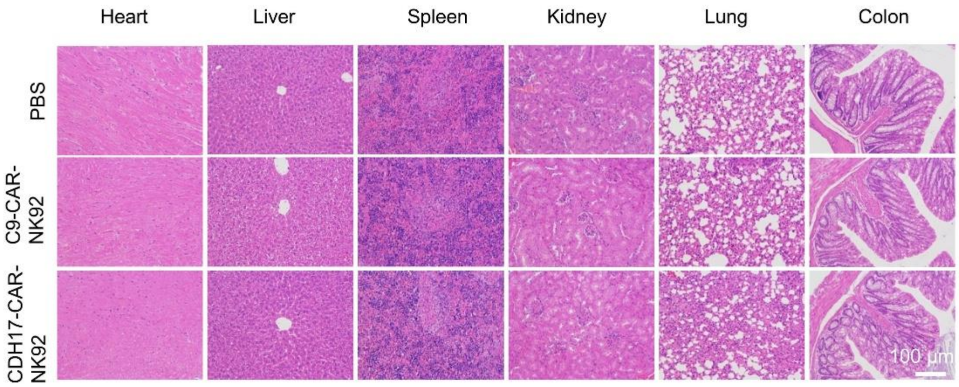

Figure S6 HE staining for IM95 burden mouse major organs from mice receiving different treatments. Scale bar: 100 µm.

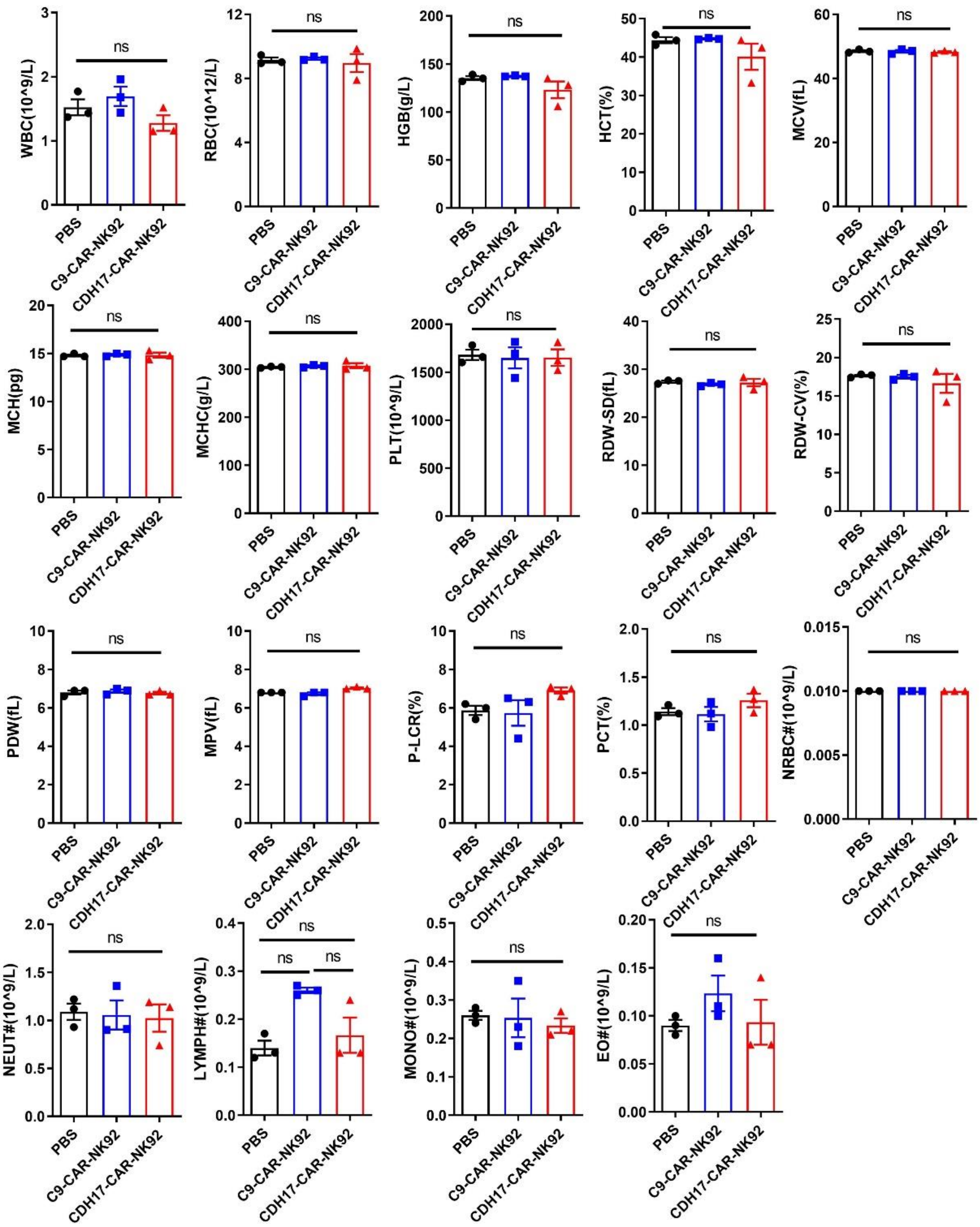

Figure S7 Whole blood routine test results of IM95-burden mouse white blood cell (WBC), red blood cell (RBC), hemoglobin (HGB), hematocrit (HCT), mean corpuscular volume (MCV), mean corpuscular hemoglobin (MCH), mean corpuscular hemoglobin concentration (MCHC), platelet count (PLT), red blood cell volume distribution width (RDW), platelet distribution width (PDW), mean platelet volume (MPV), platelet ratio (P-LCR), plateletcrit (PCT), normal red blood cell (NRBC), neutrophils (NEUT), lymphocytes (LYMPH), monocytes (MONO) and eosinophils (EO). Data are presented as mean ± SEM. The differences for each group were analyzed by one-way ANOVA analysis. ns, no significance.

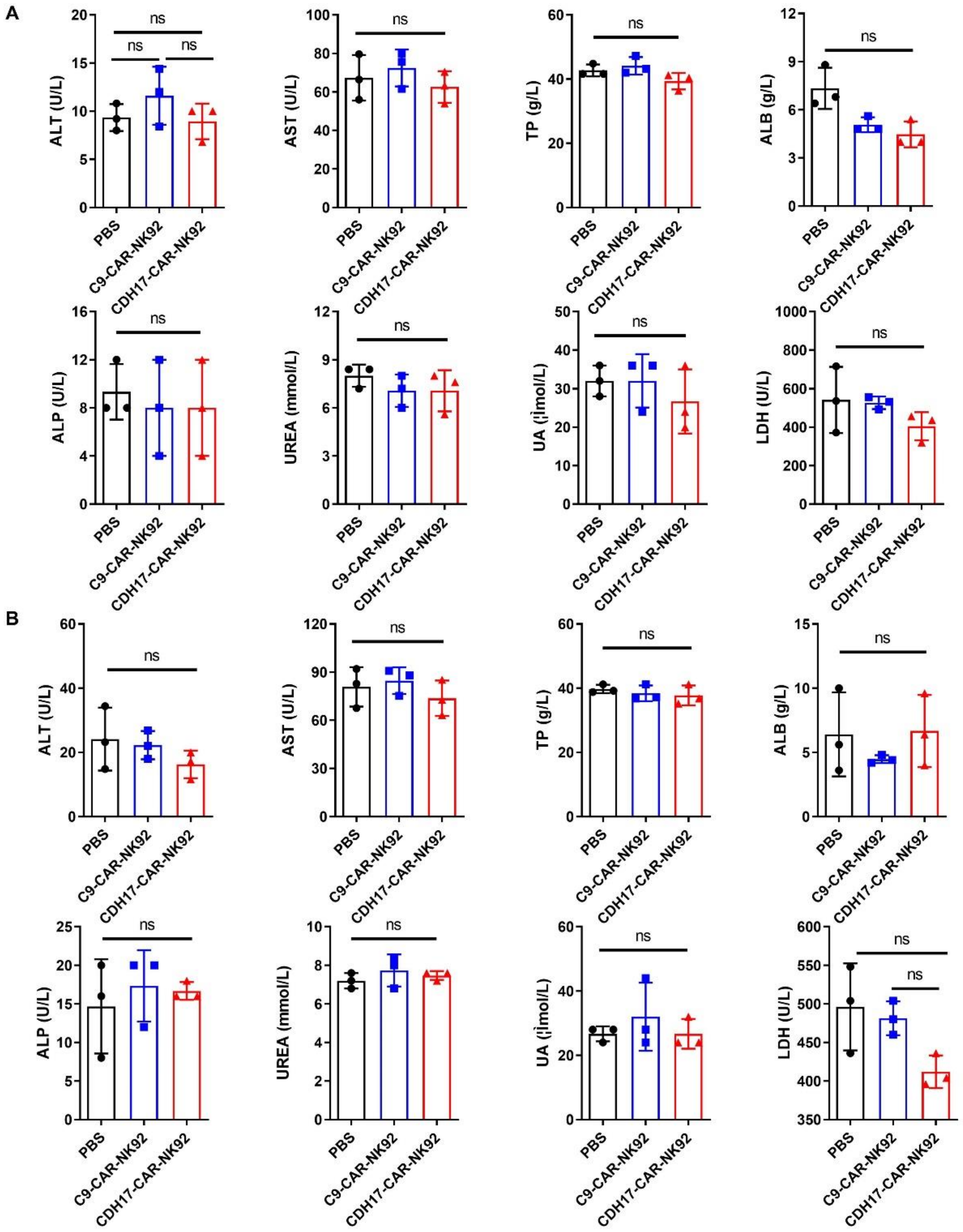

Figure S8 Serum biochemical test results of IM95 (A) and ASPC1 (B) burden mouse alanine aminotransferase (ALT), aspartate aminotransferase (AST), albumin (ALB), lactate dehydrogenase (LDH), alkaline phosphatase (ALP), urea (UREA), urine microalbumin (U-mALB) and albumin/globulin ratio (A/G). The differences for each group were analyzed by one-way ANOVA analysis. Data are presented as mean ± SEM. ns, no significance.

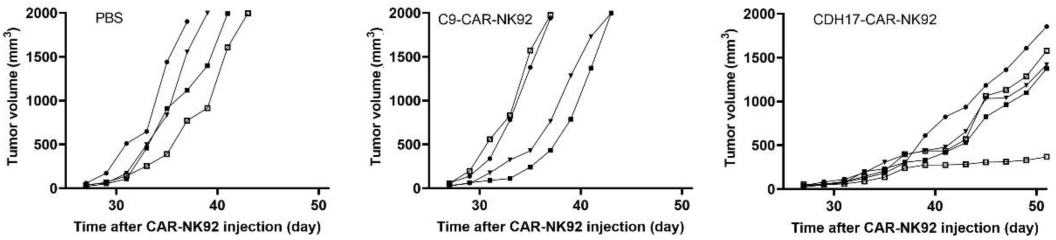

Figure S9 Individual tumor growth curves of PDX-burden mouse from each group with different treatments (n = 4–5).

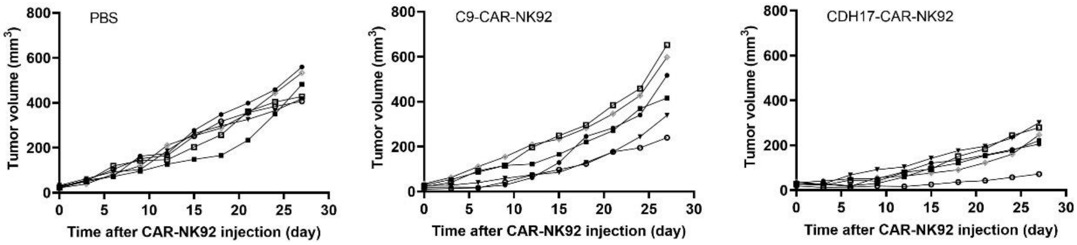

Figure S10 Individual tumor growth curves of ASPC1-burdened mouse from each group with different treatments (n = 6).

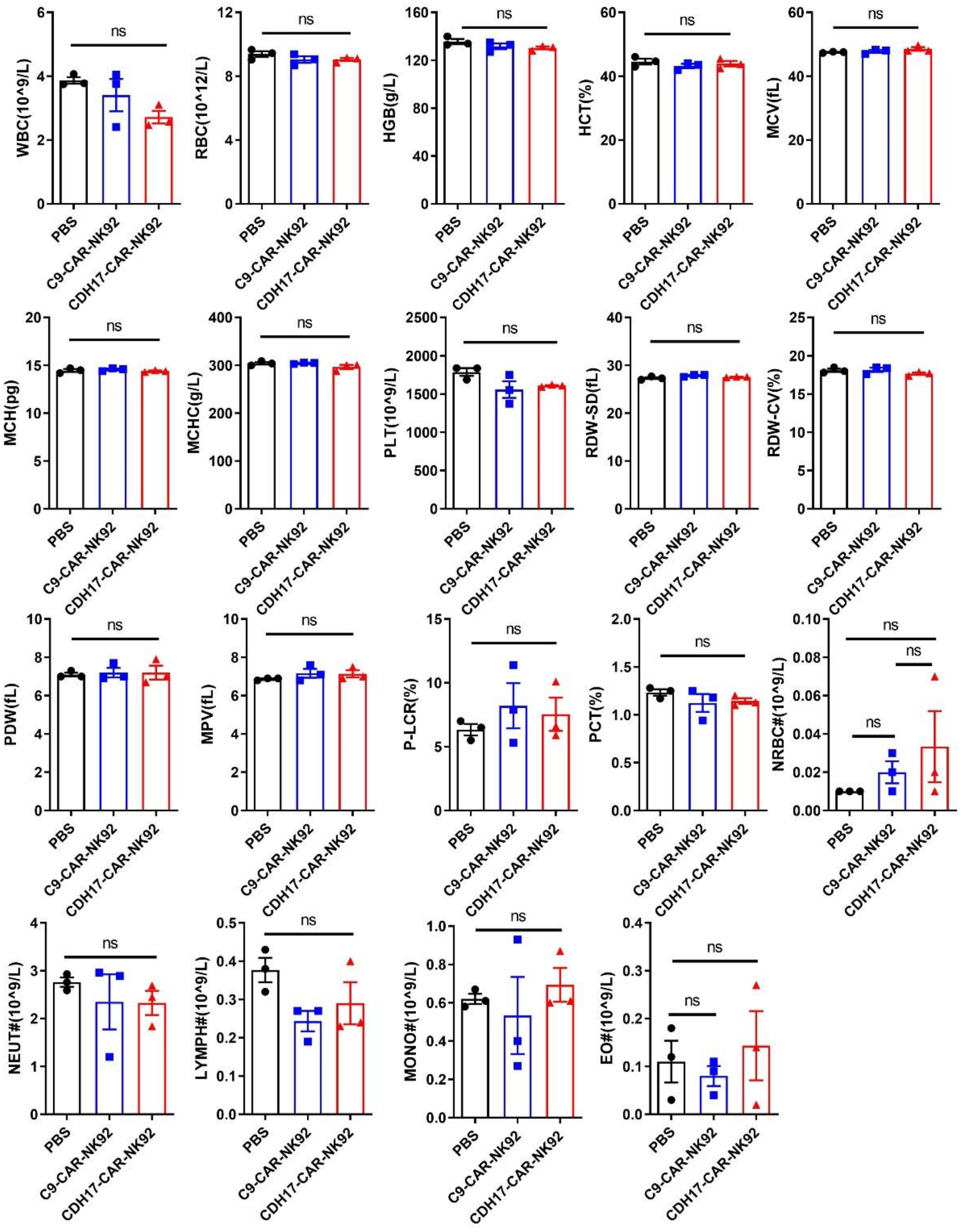

Figure S11 Whole blood routine test results of ASPC1-burden mouse white blood cell (WBC), red blood cell (RBC), hemoglobin (HGB), hematocrit (HCT), mean corpuscular volume (MCV), mean corpuscular hemoglobin (MCH), mean corpuscular hemoglobin concentration (MCHC), platelet count (PLT), red blood cell volume distribution width (RDW), platelet distribution width (PDW), mean platelet volume (MPV), platelet ratio (P-LCR), plateletcrit (PCT), normal red blood cell (NRBC), neutrophils (NEUT), lymphocytes (LYMPH), monocytes (MONO) and eosinophils (EO). Data are presented as mean ± SEM. The differences for each group were analyzed by one-way ANOVA analysis. ns, no significance.

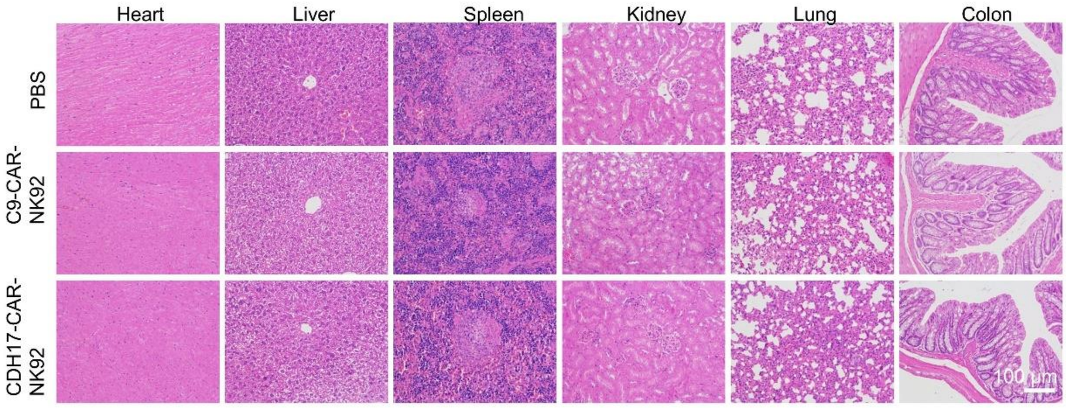

Figure S12 HE staining for ASPC1 burden mouse major organs from mice receiving different treatments. Scale bar 100 μm.

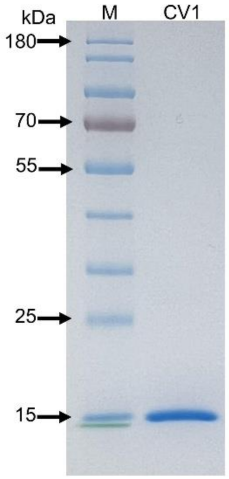

Figure S13 The purified CV1 was separated by SDS-PAGE and stained with Coomassie Blue.

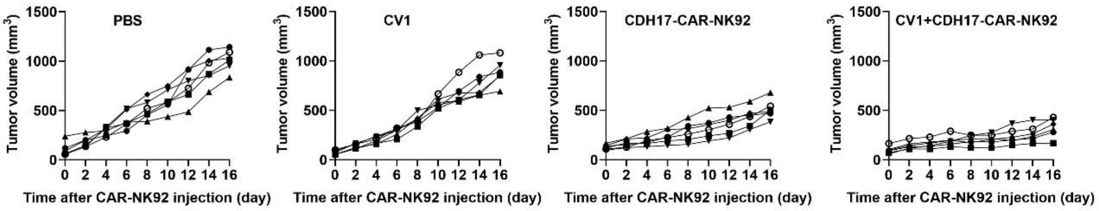

Figure S14 Individual tumor growth curves of ASPC1-burden mouse from each group with different treatments (n = 6).

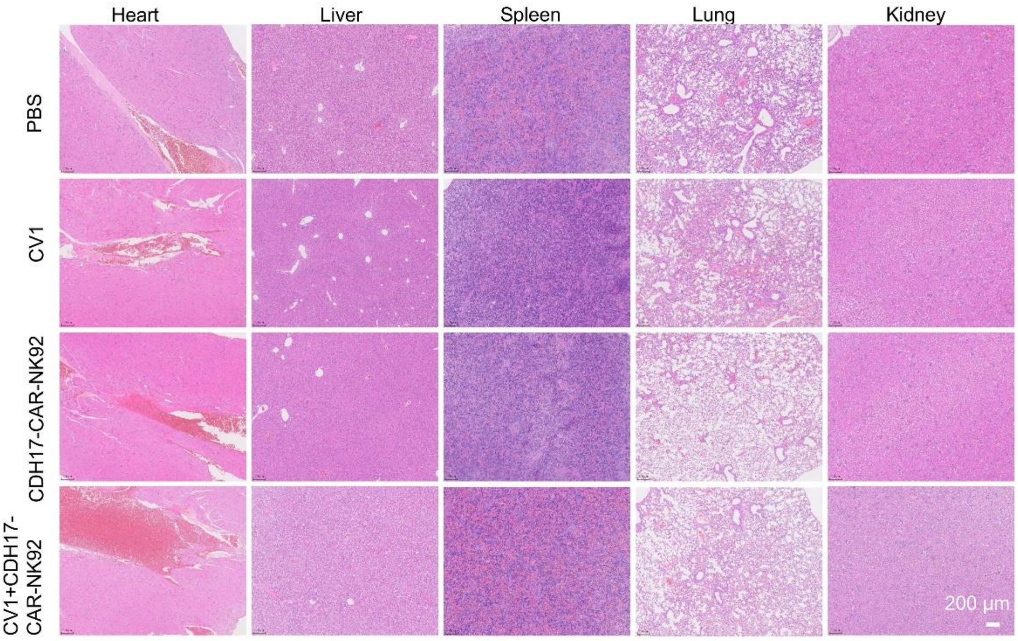

Figure S15 HE staining of vital organs in an experiment combining CDH17-targeted CDH17-CAR-NK92 cell therapy with CV1 in the ASPC1 mouse model. Scale bar 200 μm.

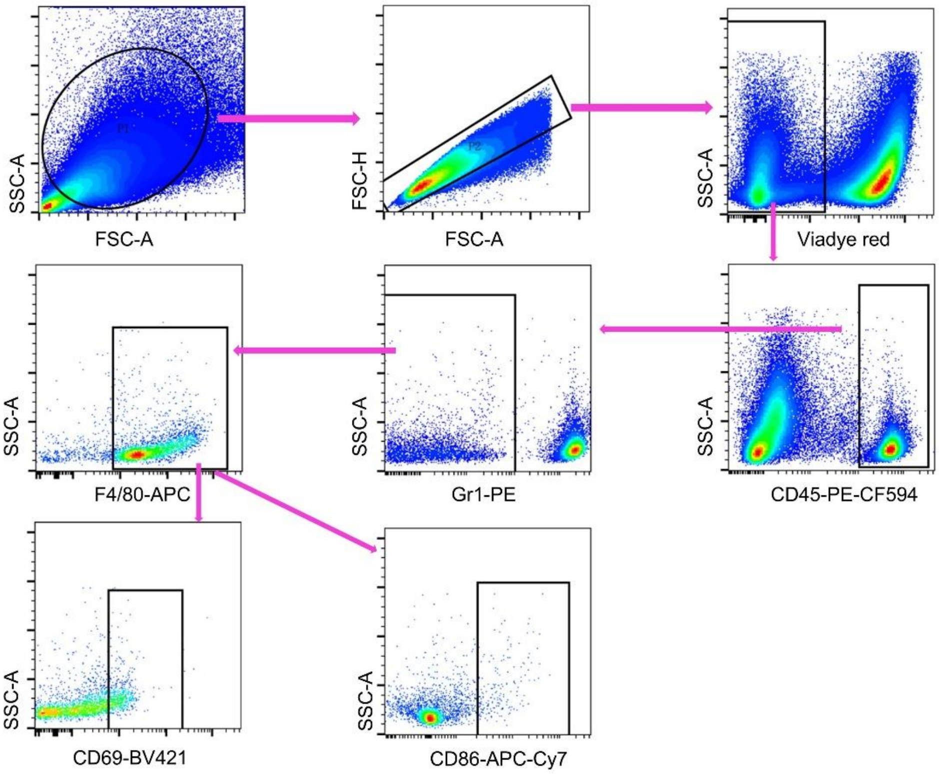

Figure S16 Gate strategy for flow cytometric analysis of macrophage activation markers CD69 and CD86 in the tumor microenvironment after different treatments.

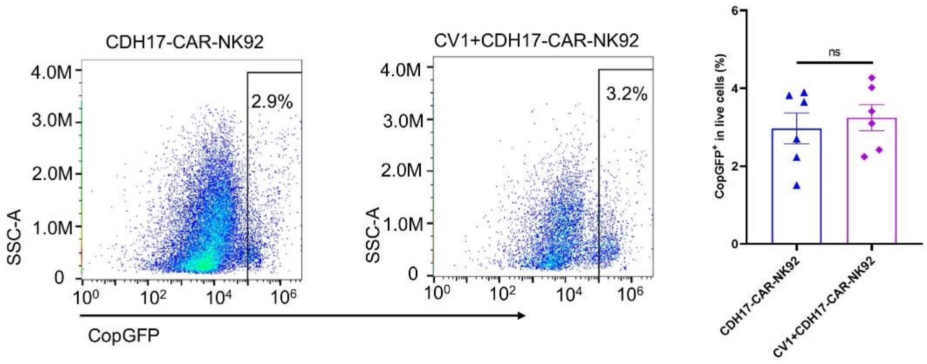

Figure S17 Flow cytometric analysis of CDH17-CAR-NK92 cell number in ASPC1 tumors treated with combinational modalities (n = 6). ns, no significance. The difference was analyzed by Student's t-test. Data are presented as mean ± SEM. ns, no significance.
